# HIF-1α switches the functionality of TGF-β signaling via changing the partners of smads to drive glucose metabolic reprogramming in non-small cell lung cancer

**DOI:** 10.1186/s13046-021-02188-y

**Published:** 2021-12-20

**Authors:** Yiwei Huang, Zhencong Chen, Tao Lu, Guoshu Bi, Ming Li, Jiaqi Liang, Zhengyang Hu, Yuansheng Zheng, Jiacheng Yin, Junjie Xi, Zongwu Lin, Cheng Zhan, Wei Jiang, Qun Wang, Lijie Tan

**Affiliations:** Department of Thoracic Surgery, Zhongshan Hospital, Fudan University, No. 180, Fenglin Road, 200032 Shanghai, China

**Keywords:** TGF-β/Smad signaling pathway, HIF-1α, Metabolic reprogramming, Cell cycle

## Abstract

**Background:**

Most cancer cells have fundamentally different metabolic characteristics, particularly much higher glycolysis rates than normal tissues, which support the increased demand for biosynthesis and promote tumor progression. We found that transforming growth factor (TGF)-β plays a dual function in regulating glycolysis and cell proliferation in non-small cell lung cancer.

**Methods:**

We used the PET/MRI imaging system to observe the glucose metabolism of subcutaneous tumors in nude mice. Energy metabolism of non-small cell lung cancer cell lines detected by the Seahorse XFe96 cell outflow analyzer. Co-immunoprecipitation assays were used to detect the binding of Smads and HIF-1α. Western blotting and qRT-PCR were used to detect the regulatory effects of TGF-β and HIF-1α on c-MYC, PKM1/2, and cell cycle-related genes.

**Results:**

We discovered that TGF-β could inhibit glycolysis under normoxia while significantly promoting tumor cells’ glycolysis under hypoxia *in vitro* and *in vivo*. The binding of hypoxia-inducible factor (HIF)-1α to the MH2 domain of phosphorylated Smad3 switched TGF-β function to glycolysis by changing Smad partners under hypoxia. The Smad-p107-E2F4/5 complex that initially inhibited c-Myc expression was transformed into a Smad-HIF-1α complex that promoted the expression of c-Myc. The increased expression of c-Myc promoted alternative splicing of PKM to PKM2, resulting in the metabolic reprogramming of tumor cells. In addition, the TGF-β/Smad signal lost its effect on cell cycle regulatory protein p15/p21. Furthermore, high expression of c-Myc inhibited p15/p21 and promoted the proliferation of tumor cells under hypoxia.

**Conclusions:**

Our results indicated that HIF-1α functions as a critical factor in the dual role of TGF-β in tumor cells, and may be used as a biomarker or therapeutic target for TGF-β mediated cancer progression.

**Supplementary Information:**

The online version contains supplementary material available at 10.1186/s13046-021-02188-y.

## Background

Lung cancer is the leading cause of cancer-related deaths worldwide [1]; non-small cell lung cancer (NSCLC) accounts for 85% of the histological types of lung cancer. TGF-β plays an important role in the progression of NSCLC. TGF-β initiates signal transduction through the phosphorylation and subsequent nuclear translocation of Smad2 and Smad3 [2], which form a complex with Smad4 to regulate the transcription of target genes, such as c-Myc [3] and cyclin-dependent kinase inhibitor p21^Cip1^(p21) [4, 5], p27^kip1^ (p27), and p15^Ink4b^ [6]. In normal epithelial cells and early tumor cells, TGF-β usually inhibits cell proliferation by regulating cell cycle-related genes. However, in advanced tumors, TGF-β can promote proliferation, invasion, and metastasis of tumor cells [2, 7]. Studies have revealed that this is due to the mutation of key signaling molecules in the process of TGF-β signal transduction, leading to a loss of its antitumor effects in tumor cells [8, 9]. In tumor cells with an intact TGF-β signaling pathway, it was found that TGF-β could still promote tumor progression. It is believed that the Smad partners change during TGF-β signaling, leading to loss of the original inhibitory function of TGF-β against specific genes that promote tumor progression [6, 10].

Transforming growth factor (TGF)-β1 has been reported to induce a switch of tumor-associated fibroblasts from oxidative phosphorylation to aerobic glycolysis [11]. A common tumorigenesis mechanism is the reprogramming of energy metabolism to aerobic glycolysis [11]. During the epithelial-mesenchymal transition induced by TGF-β, changes in the glucose metabolism of tumor cells have been widely observed, such as by promoting the expression of Glut1 and PFKFB3 to increase cellular glucose uptake [12–14]. TGF-β can also induce PFKFB3 and HK2 expression by activating p38 MAPK, and PI3K/Akt signaling pathways and mediate the reprogramming of glioma cells [15]. Furthermore, it was reported that upon TGFβ-induced EMT of lung adenocarcinoma cells, the expression of PKM2 increases [16]. This means that the regulation of glycolysis by TGF-β in tumor cells may be achieved by regulating the expression of PKM2.

The PKM gene encodes a primary transcript, which contains two mutually exclusive exons, namely exon 9 and exon 10, which encode two different isoforms PKM1 and PKM2, respectively [17]. PKM1 is expressed in terminally differentiated tissues to promote oxidative phosphorylation. In contrast, PKM2 is highly expressed in embryos and cancer cells, an allosteric isomer, and exhibits a dimer with a low affinity for PEP [18]. It has been reported that the ratio of PKM2/PKM1 in a variety of cancers has increased, and it is closely related to the shortening of the overall survival of cancer patients [19, 20]. The splicing factors of heterogeneous nuclear ribonucleoprotein (hnRNP) A1/2 and polypyrimidine bundle binding (PTB) proteins drive alternate splicing of PKM pre-mRNA by selectively including exon 10 and repelling exon 9 [21, 22]. Many factors can also drive the expression of PKM2 gene, especially HIF-1α plays an important role [23].

HIF-1α is widely expressed in human tumors, and higher expression has been observed in advanced tumors with increased tumor volume. HIF-1α is highly expressed in the hypoxic environment. Hypoxia is a vital tumor microenvironment characteristic resulting from excessive oxygen consumption and insufficient vascular supply in rapidly growing tumors [24]. Reactions to hypoxia are induced by a series of precise regulatory mechanisms when oxygen density is decreased in tumor cells, mediated by hypoxia-inducible factor (HIF)-1, a heterodimer composed of α- and β-subunits [25]). HIF-1α promotes tumor progression by regulating glycolysis, angiogenesis, and cell cycle progression of tumor cells [26, 27]. Although it is known that both TGF-β and HIF-1α can regulate the glucose metabolism of tumor cells, the effect of high expression of HIF-1α induced by hypoxia on the regulation of TGF-β/Smad signaling is poorly understood.

## Methods

### Cell lines and cell culture

Cell lines (A549, H1299, and 293T) were purchased from the cell bank of the Chinese Academy of Sciences. Cells were cultured in DMEM high glucose medium (Hyclone, Logan, UT, USA) with 10% fetal bovine serum (Hyclone), 100 U/ml penicillin, and 100 U/ml streptomycin. Cells were incubated in a 5% (vol/vol) CO_2_/95% (vol/vol) air incubator at 37 °C. Hypoxia-cultured cells were cultured in a 1% O_2_/5% (vol/vol) CO_2_/balance N_2_ flushed modular incubator and incubated at 37 °C. For *in vitro* detection of TGF-β function, following removal of the original medium, cells were treated with fresh medium containing 5 ng/ml TGF-β (+) or vehicle (-) for 4 h.

### Antibodies and reagents

TGF-β1 was purchased from T&L Biological Technology (#TL-643, Beijing, China). The antibodies used in this study are listed in Supplementary Table 1. According to the instructions, the concentration of the primary antibody was 1:1000 ~ 2000. The antibody was diluted with primary antibody diluent (P0256, beyotime, Shanghai, China). HRP labeled goat anti-mouse and goat anti-rabbit IgG (H + L) IgG (1:4000, a0208, beyotime, Shanghai, China).

*Plasmids.*

Deletion mutants Smad3-MH1 (Smad3 amino acids 1-136), Smad3-LC (Smad3 amino acids 137-425), and Smad2-MH2 (Smad3 amino acids 232-425) were generated by PCR. The Smad3 cDNAs were subcloned into derivative vectors of pRK5F (Flag-tag) for mammalian expression. Lentiviral constitutive expression plasmids for Flag-tagged Smad4, Smad2, E2F4, E2F5, and p107 were constructed by subcloning into WPI-Puro-iRES. The Smad2/3 shRNA plasmid hU6-MCS-CMV-ZsGreen 1-PGK-Puro, c-Myc overexpression plasmid CMV-MCS-PGK-Blasticidin, as well as their empty vector were constructed by the Hanyin company (Shanghai, China).

### Transfection of shRNA, siRNA and overexpression RNA

Six different short hairpin RNAs (shRNAs) (Smad2-shRNA#1, Smad2-shRNA#2, Smad3-shRNA#1, Smad3-shRNA#2, HIF-1α-shRNA#1, and HIF-1α-shRNA#2) targeting human Smad2, Smad3, and HIF-1α were designed and cloned into puromycin and blasticidin resistant lentivirus vectors obtained from the Hanyin company (Shanghai, China). Two different cell lines were transfected and screened with puromycin and blasticidin. Similarly, four lentiviral vector-mediated overexpression RNA (c-Myc-OE#1, c-Myc-OE#2, HIF-1α mut #1, HIF-1α mut #2) were designed for overexpression of human c-Myc and HIF-1α. Sequences of shRNAs and controls are provided in Supplementary Table 1. Two siRNA for knockdown of the PKM2 were constructed with plasmid (siPKM2 #1, siPKM2 #2).

### RNA preparation and quantitative real-time polymerase chain reaction (qRT-PCR)

According to the manufacturer’s RT-PCR protocol, total RNA of cells was extracted using TRIzol reagent (Invitrogen, Carlsbad, CA, USA). cDNA template was synthesized using PrimeScript RT Reagent Kit (TaKaRa, Tokyo, Japan) and qRT-PCR was conducted using SYBR Premix Ex Taq (Takara) in the Applied Biosystems system 7500 (Thermo Fisher Scientific, Waltham, MA, USA). The 2^−ΔΔCT^ method was used to perform relative quantification of mRNA analysis using β-actin as an endogenous control. The sequences of primers used for qRT-PCR are shown in Supplementary Table 1. All of the primers were synthesized by Sangon Biotech (Shanghai, China). The primer sets used in PCR were shown in the attached table. The PCR primer sets for PKM1 and PKM2 were primers that specifically amplify exon 9 and exon 10 fragments.

### DNA pull-down assay

Biotinylated HRE oligonucleotide probes were used for DNA pull-down assay. The biotin-labeled HRE oligonucleotide probes were fixed with streptavidin magnetic beads (Solarbio LIFE SCIENCES) and washed with DNA buffer I (10 mM Tris HCl (pH 7.5), 1 mM EDTA, 1 mM NaCl, 0.01-0.1% Tween-20) according to the instructions. HEK293T cells were incubated with biotinylated SBE oligonucleotides for 30 min. The precipitate was washed thoroughly with cold PBS, boiled in SDS sample buffer, and analyzed by Western blotting.

### Western blotting

Nuclear protein and cytoplasmic protein extraction kit (P0027, beyetime, Shanghai, China) were used to extract the nuclear protein and cytoplasmic protein by following the product description. Total cellular protein was extracted from cells using RIPA buffer (Beyotime, Shanghai, China) containing a protease and phosphatase inhibitor cocktail and quantified using the enhanced BCA Protein Analysis Kit (Yisheng, Shanghai, China). Then, the proteins were separated by SDS-PAGE and transferred onto polyvinylidene fluoride membranes (Merck Millipore, Burlington, MA, USA). After blocking with 5% non-fat milk, proteins were probed with the indicated primary antibody followed by the appropriate horseradish peroxidase-conjugated secondary antibody. Protein bands were detected using BeyoECL Moon Chemiluminescence Reagent (Beyotime).

### Cell proliferation assay

Cells in logarithmic growth were inoculated into a black 96-well plate (Life Science, Oneonta, NY, USA) in a suspension of 100 ul/well with a total of 3000 cells. The cells were incubated at 37 °C for 4, 24, 48, 72, and 96 h and cell proliferation were measured with the Enhanced Cell Counting Kit-8 Viability Assay Kit (10 ul/well) (Beyotime) at each timepoint. The optical density (absorbance) at 450 nm was measured with the microplate reader of the multi-wavelength measurement system (Flexstation® 3, Molecular Devices (MD), USA).

### Plate clone formation experiment

Cells in logarithmic growth were digested into a single cell suspension for counting. The cell suspension was inoculated in triplicate into a six well plate at a density of 300 cells/well and cultured in the above-mentioned normoxic and hypoxic environments for 2 weeks. Each well was washed with phosphate-buffered saline. Clones were fixed with 4% methanol for 15 min and stained with 1% purple crystal for 20 min. Viable colonies with a diameter ≥ 0.2 mm were counted and the clone formation rate was calculated.

### Immunofluorescence

The cells were cultured and treated on the cell climbing plate of the six-well plate. We used paraffin sections of Lung cancer tissue and adjacent tissue for tissue immunofluorescence staining. Incubated with QuickBlock^TM^ immune staining blocking solution (4% formaldehyde), 0.5%Triton X-100,5% Bovine Serum Albumin (PBS), first Antibody, Labeling Goat anti-mouse IgG (H + L) with Alexa fluor 488, labeling Goat anti-rabbit IgG (H + L) with Alexa fluor 647 and DAPI, respectively. Immunofluorescence images were collected by the Zeiss LSM710 confocal microscope (Germany).

### Tissue hypoxia detection

The Image-iT™ Green Hypoxia Reagent is an end-point assay reagent, whose signal increases with reduced oxygen levels, but is not reversible. Flowed by the instruction of the reagent, we detected the oxygen concentration situation of the subcutaneous tumor.

### Determination of glucose, lactate, ECAR, and OCR levels

The extracellular acidification rate (ECAR) and cellular oxygen consumption rate (OCR) were detected by the Seahorse XFe96 cell outflow analyzer (SeahorseBioscience, Billerica, MA, USA). The cells were implanted into 96-well plates at a density of 5 × 10^5^ cells per well. After the basic measurements were recorded, ECAR was detected by adding glucose (25 mM), oligomycin (5 µM), and 2-deoxyglucose (2 mM) to the microwell plate of Seahorse XFe96. In the determination of OCR, oligomycin (5 µM), p-trifluoromethoxycarbonyl cyanophenylhydrazone (FCCP; 1.5 µM), and mitochondrial complex I inhibitor rotenone + mitochondrial complex III inhibitor antimycin A (Rote+AA; 5 µM) were added in turn. FCCP is an uncoupling agent that disrupts the proton gradient and mitochondrial membrane potential. It is the second compound injected after oligomycin. The result of the addition is that the electron flow through the ETC is not restricted, and the oxygen consumption of the complex IV reaches a large. The OCR stimulated by FCCP can be used to calculate the reserve breathing capacity, which is defined as the difference between the major respiration and the basic respiration. Spare breathing capacity measures the ability of cells to respond to increased energy demand or stress. 2-deoxyglucose is a glucose analogue that inhibits glycolysis by competitively binding to the first enzyme in the glycolysis pathway, glucose hexokinase. The resulting decrease in PER provides qualitative confirmation that the PER produced before the addition of 2-DG mainly comes from glycolysis. Mitochondrial electron transport chain inhibitor to inhibit mitochondrial oxygen consumption and thus inhibit CO2 sourced protons. Glucose uptake colorimetric analysis kit (Biovision, Mil-Pitas, California, USA) and lactic acid analysis kit II (Biovision) were used to detect the glucose consumption and lactate production of lung cancer cells according to the manufacturer’s instructions.

### Reporter luciferase assay

The recombinant plasmids containing c-Myc promoter-Luc/Rluc and corresponding mutation sites were prepared and transfected into A549 and H1299 cells. Cells were treated with 5 ng/ml TGF-β or vehicle control for 24 h. The microplate reader of the multi-wavelength measurement system (Flexstation® 3, Molecular Devices (MD), USA) was used to detect the fluorescence intensity.

### Co-immunoprecipitation assays

In the co-immunoprecipitation experiment, the cells were cultured in a 10 cm cell culture dish, washed with PBS, and 1ml of cell lysate was added. Take 100ul protein and add 0.2-2 ug of primary antibody for immunoprecipitation. After shaking slowly overnight at 4 °C, add 40 ul of fully resuspended Protein A+G Agarose, shaking slowly at 4 °C for 3 h. Centrifuge at 2500 rpm (about 1000 g) for 5 min, or centrifuge at high speed instantaneously, and carefully aspirate the supernatant. Wash the precipitation 5 times with PBS. After finishing the last wash, remove the supernatant and add 40 µl 1X SDS-PAGE loading buffer and vortex to resuspend the precipitation. After treatment at 100 °C or boiling water bath for 3-5 min, take part or all of the samples for Western blotting.

### Animal experiments

All animals received standard care, and animal procedures were approved by the Institutional Review Committee of Zhongshan Hospital, Fudan University (Shanghai, China) (approval number: IBCB-SPF1314). Male athymic nude mice (BALB/cASlac-nu) were all bred in the specific pathogen-free background. For the xenotransplantation tumor growth test, 1.0 × 10^7^ cells were injected subcutaneously into each mouse. A549 cells transfected with shRNA targeting Smad3 and HIF-1α, as well as their controls were injected into the groin of the hind limb of mice. Tumor size was measured once a week. After 45 days, the surviving nude mice were sacrificed by cervical dislocation. After mice sacrifice, the tumors were separated and weighed. When one of the following signs of disease was observed, the animals were sacrificed: obvious tumor ulcer (> 0.5 cm); serious injury; unable to move or eat. The PET/MRI imaging system for small animals (United Well Technologies (China) Limited, nanoScan® PET/MRI, China) was used to observe the glucose metabolism of subcutaneous tumor in nude mice. The Optical imaging system for small animals in vivo (IVIS Spectrum, PerkinElmer, USA) was used to detect the changes in tumor size.

### Human samples

Human surgical samples were obtained from the Department of Thoracic Surgery, Zhongshan Hospital, Fudan University (Shanghai, China) between October 2008 and September 2010. By consulting the case information of related patients, we searched for and collected postoperative frozen non-small cell lung cancer tissue samples of 121 patients who underwent PET-CT before surgery. Then, corresponding lung cancer tissue chips were then manufactured. We followed up with these patients every six months. All of the human tissues used in this study were acquired from patients who provided informed consent; patients remained anonymous when their clinical information was used. The Institutional Review Committee of Zhongshan Hospital, Fudan University (Shanghai, China) approved the use of the clinical information and human tissue samples in this study (approval number: IBCB-0213).

### Bioinformatics Analysis

Glycolysis score was based on the expression abundance of glycolysis-related genes in TCGA data. Based on this score, the non-small cell lung cancer cases in the TCGA data were divided into two groups with high and low glycolysis scores and search for genes that were significantly differentially expressed between the two groups. Then construct the expression regulation network of transcription factors and glycolysis-related genes according to the correlation of these differential genes.

### Statistical analysis

All data were expressed as mean±standard deviation (SD). The differences between two or more groups were analyzed by Student’s t-test and analysis of variance. Pearson’s x^2^ test was used to analyze the relationship between the expression level of genesand clinicopathological characteristics. Kaplan-Meier analysis and log-rank test were used to assess differences in patient survival. Statistical analysis was performed using SPSS software (SPSS package v24.0, Inc., Chicago, Illinois, USA) and GraphPad Prism 7.0 (GraphPad Software, La Jolla, CA, USA). The data was statistically processed: *p value<0.001, **p value<0.01, *p value<0.0001, *p value<0.05, *p value<0.01, *p value<0.05, *p value<0.0001. Bilateral p-value < 0.05 was considered to be statistically significant.

## Results

### TGF-β plays a dual function in regulating the glucose metabolism of NSCLC under normoxia and hypoxia

**Fig. 1:**
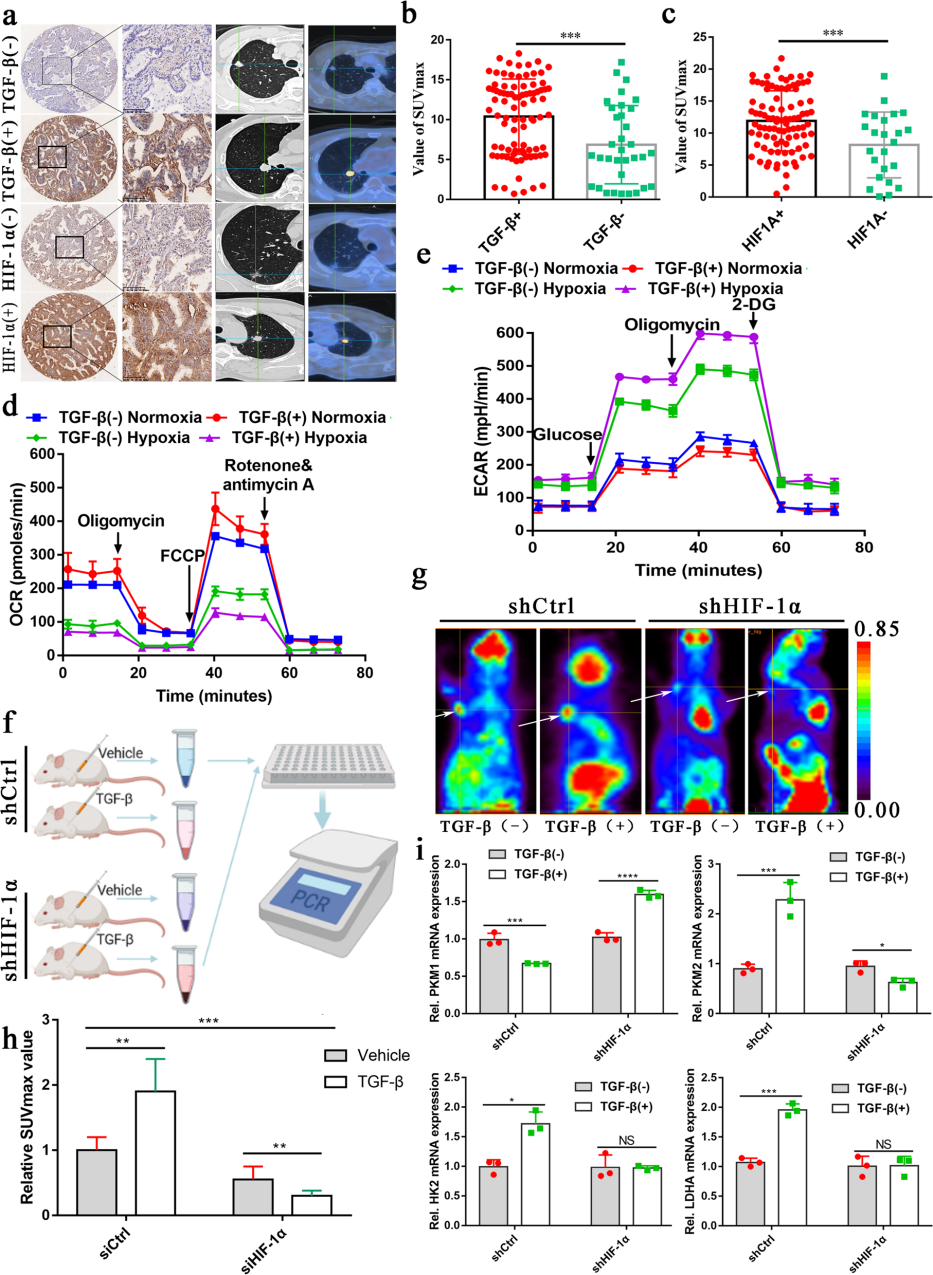
TGF-β plays a dual function in regulating the glucose metabolism of NSCLC under normoxia and hypoxia.** a**, Representative 18 F-FDG PET/CT imaging of NSCLC patients with positive or negative TGF-β expression (scale bar, 100 μm). **b, c**, SUVmax analysis was performed in TGF-β positive (n=87) and TGF-β negative (n=34) by Mann-Whitney U rank-sum test, as well as HIF-1α positive (n=94) and HIF-1α negative (n = 24) NSCLC patients. The data are shown as mean ± standard deviation (SD). **d, e**, ECAR (**e**) and OCR (**d**) showed that A549 cells were metabolically reprogrammed following treatment with 5 ng/ml TGF-β under normoxia and hypoxia for 24 h, as examined by Seahorse Extracellular Flux Analyzer XF96 assay. **f**, Experimental schematic to verify the effect of TGF-β and HIF-1α on glucose uptake of tumor cells *in vivo*. **g**, Representative typical 18 F-FDG microPET imaging of subcutaneously implanted tumor model mice. Xenograft imaging of A549 cells with knockdown of HIF-1α and control groups after TGF-β (5 ng/ml, injection once a week) and its solvent injection was indicated by the white arrow (Three nude mice per group). **h**, The relative ratios of SUVmax of A549 cells with and without HIF-1α knockdown following injection of TGF-β and vehicle. **i**, ***P < 0.001, **P < 0.01; P-values were calculated with a two-tailed *t*-test. 2-DG, 2-deoxy-D-glucose; FCCP, carbonyl cyanide 4-(trifluoromethoxy) phenylhydrazone; 18 F-FDG, 18 F-fluorodeoxyglucose; ECAR, extracellular acidification rate; OCR, oxygen consumption rate; PET, positron emission tomography; SUVmax, maximum standardized uptake value. The concentration of all TGF-β used in this part of the experiment was 5 ng/ml

By analyzing the Cancer Genome Atlas (TCGA) database data, we found that TGF-β and HIF-1α were significantly overexpressed in patients with high glycolysis scores. They were widely involved in regulating the expression of glycolysis-related genes (Supplementary Fig. 1a). PET/CT (18 F-FDG) has been extensively applied to visualize the metabolic activity of solid tumor cells. The maximum standardized uptake value (SUVmax) has been regarded as an alternative indicator for the prognosis of lung cancer (Sun et al. [28]). The maximum standardized uptake value (SUV) was calculated to quantitatively analyze tumor FDG uptake as follows: SUV = C (kBq / ml) / ID (kBq) / body weight (kg). In the formula, C is the tissue activity concentration measured by PET, and ID is the injection dose. We investigated the expression of TGF-β and HIF-1α in tissue chips derived from NSCLC patients by immunohistochemistry. We then analyzed the relationship between the imaging findings of PET/CT and the expression of TGF-β and HIF-1α (Fig. 1a). Interestingly, we found that lung cancer patients with positive TGF-β and HIF-1α expression exhibited high SUVmax (Fig. 1b, c).

Furthermore, we explored the effect of TGF-β on cell metabolism *in vitro*. We found that glucose intake and lactate production were decreased in A549 and H1299 cells cultured in medium containing TGF-β (5 ng/ml) in normoxia (See the method section ‘*cell culture*’ for details) (Supplementary Fig. 1b, c, e, g). Moreover, the extracellular acidification rate (ECAR) decreased while the oxygen consumption rate (OCR) increased in A549 cells (Fig. 1d, e), which contradicts the above results. Considering a significant difference between the solid tumor and cells cultured *in vitro* (the solid tumor is almost in a state of hypoxia (Patel and Sant [29])), we cultured the cells under hypoxia. A total turnaround metabolic phenotype was observed in both cell lines. Under normoxic conditions, after 24 h of culture with exogenous TGF-β (5 ng/ml), the glucose uptake and lactate production in A549 and H1299 cells decreased significantly. On the contrary, TGF-β obviously promoted the absorption of glucose and the production of lactate under hypoxic conditions (Supplementary Fig. 1b, c, e, g). Additionally, the ECAR increased while the OCR decreased (Fig. 1d, e). These findings indicate that TGF-β may have opposing functions in regulating the glucose metabolism of NSCLC cell lines under normoxia and hypoxia. To verify whether HIF-1α plays an essential role in this process, we subcutaneously injected A549 cells with or without HIF-1α knockdown into nude mice followed by injection with TGF-β (30 µl, 5 ng/ml) or vehicle control once a week (Fig. 1f). In the fifth week of PET imaging, we observed that the SUVmax value of A549 cells without HIF-1α knockdown was significantly increased after injection of TGF-β. By contrast, the SUVmax of A549 cells with HIF-1 knockdown decreased significantly after TGF-β injection (Fig. 1 g, h, Supplementary Fig. 1d). We then extracted total RNA from subcutaneous tumor tissue and detected the mRNA expression of several critical genes related to glycolysis by qRT-PCR. The qRT-PCR results revealed contradictory changes in PKM1 and PKM2 expression (Fig. 1i, Supplementary Fig. 1f). In addition, some key glycolysis genes such as the mRNA level of HK2, ENO1, and LDHA increased after treated by TGF-β (5 ng/ml). However, when HIF-1α was knocked down, TGF-β did not change the mRNA expression of these genes (Supplementary Fig. 1Cite ESM.f).

### TGF-β promotes glycolysis of NSCLC cells under hypoxia by increasing the PKM2/PKM1 ratio through the canonical TGF–β/SMAD signaling pathway

**Fig. 2:**
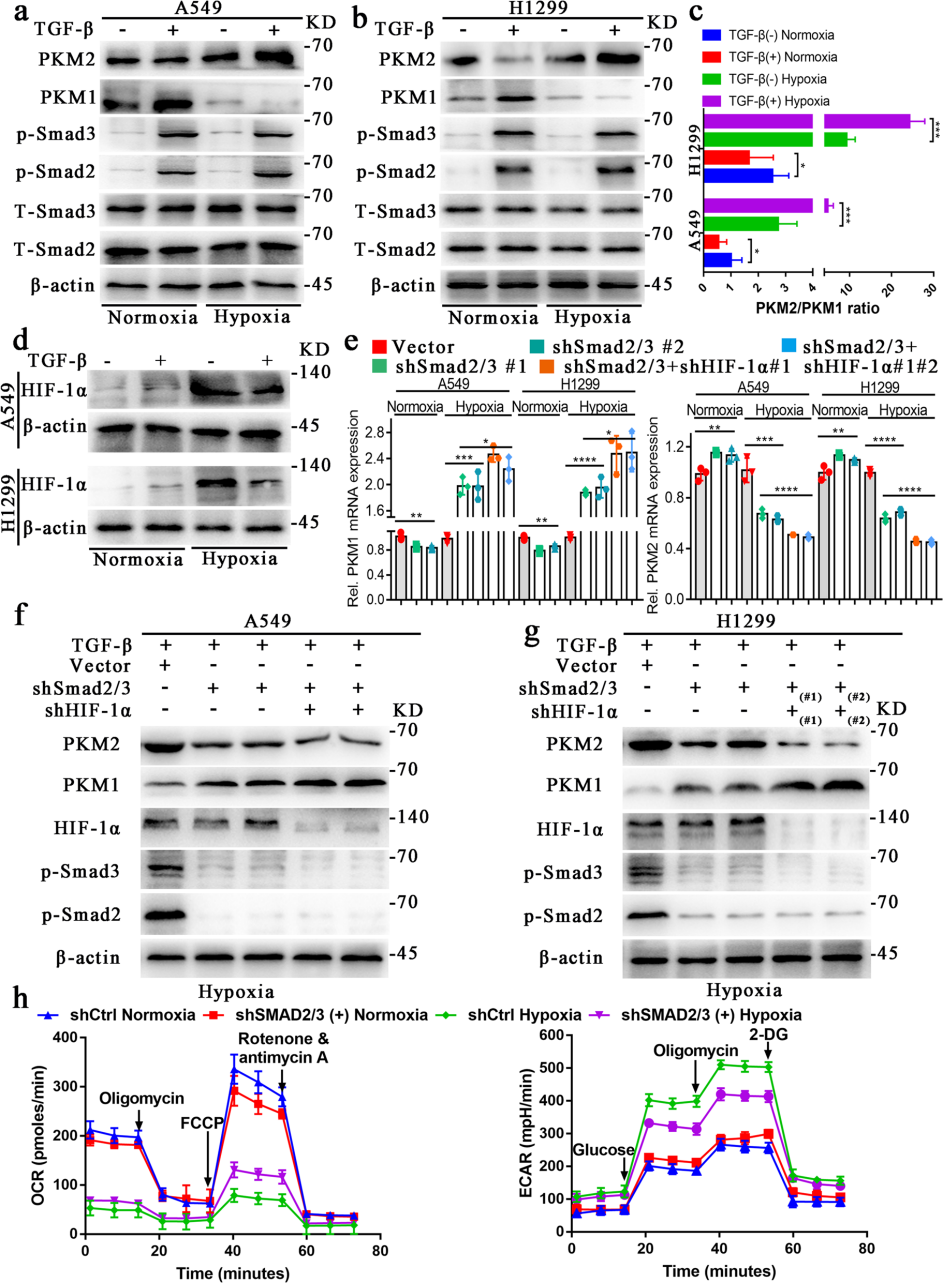
TGF-β promotes glycolysis of NSCLC cells under hypoxia by increasing the PKM2/PKM1 ratio via canonical TGF–β/SMAD signaling. **a, b**, Following the addition or loss of TGF-β in A549 and H1299 cells, total proteins were extracted and western blot analysis with the indicated antibodies was performed. **c**, The PKM2/PKM1 ratio was increased dramatically in hypoxia while decreased in normoxia when treated with TGF-β in A549 and H1299 cells. **d**, Following the culture of A549 and H1299 cells with and without TGF-β (5 ng/ml) in normoxic and hypoxic environments, the expression of HIF-1α was detected by western blotting. **e**, When Smad2/3 was knocked down, the relative RNA expression of PKM1 and PKM2 in A549 and H1299 cells (Treated with TGF-β) showed opposite changes in normoxic and hypoxic conditions. This trend of change was more significant after knocking down HIF-1α at the same time. **f, g**, Total proteins were extracted from A549 and H1299 cells with or without Smad2/3 and HIF-1α knockdown and western blot analysis was performed with the indicated antibodies. **h**, ECAR and OCR reflect the influence of p-Smad2/3 and oxygen concentration on metabolic reprogramming, as examined by Seahorse Extracellular Flux Analyzer XFe96 assay. The concentration of all TGF-β used in this part of the experiment was 5 ng/ml

We cultured the A549 and H1299 cells in normoxic and hypoxic environments (See the method section ‘cell culture’ for details) with DMEM medium containing TGF-β (5 ng/ml) or vehicle for 6 h. The western blot results showed that the expression of phosphorylated (p)-Smad2 and Smad3 increased observably when cells treated with TGF-β. In contrast, total Smad2 (T-Smad2) and total Smad3 (T-Smad3) expression were decreased. The expression of PKM2 was significantly upregulated under hypoxia, while the expression of PKM1 was decreased (Fig. 2a, b). The PKM2/PKM1 ratio was increased dramatically in hypoxia compared to normoxia (Fig. 2c).

To test whether TGF-β promotes glycolysis of cells by canonical TGF-β/SMAD signaling, we knocked down Smad2 and Smad3 (Supplementary Fig. 2a, b, and d) in A549 and H1299 cells because these two genes are essential for TGF-β signaling (Massagué [2]). After knocking down smad2/3 under hypoxia, the RNA expression level and protein expression level of PKM2 decreased; while for PKM1, the RNA expression level and protein expression level increased (Fig. 2f, g, Supplementary Fig. 2a, b, and d).

Under normoxia, the combination of hydroxylated HIF-1α and E3 ubiquitin ligase complex results in the rapid degradation of HIF-1α (Haase [30]). Our results showed little expression of HIF-1α in A549 and H1299 cells under normoxia, and they were not influenced by TGF-β (Fig. 2d). Conversely, during hypoxia, HIF-1α was not ubiquitinated and degraded (Jaakkola et al. [31]). Therefore, we hypothesized that the interaction between HIF-1α and the TGF-β/Smad signaling pathway might explain the opposing roles of TGF-β in NSCLC cell lines under normoxia and hypoxia. Knockdown of HIF-1α or Smad2/3 decreased TGF-β-induced PKM2 mRNA and protein expression but increased PKM1 mRNA and protein expression in A549 and H1299 cells under hypoxia. Simultaneously knocking down smad2/3 and HIF-1α has a more significant effect on the regulation of PKM1/2 expression than knocking down Smad2/3 alone (Fig. 2e, f, g). However, in the normoxic environment, we found that the expression of PKM2 increased significantly while the expression of PKM1 decreased significantly (Fig. 2e). These results suggest that HIF-1α induced by hypoxia plays a critical role in regulating PKM2 and PKM1 expression.

We then investigated the effect of Smad2/3 knockdown on glycolysis under hypoxia and normoxia. The OCR increased, but the EACR decreased in A549 cells with knockdown of Smad2/3 under hypoxia. However, opposite results were observed in normoxia (Fig. 2 h). Similarly, tumor cells’ glucose uptake and lactate production with and without Smad2/3 knockdown also showed opposite results under normoxic and hypoxic conditions (Supplementary Fig. 2e–h). These results indicate that TGF-β promotes the glycolysis of NSCLC cells, and this effect can be blocked by knockdown of Smad2 and Smad3.

### Interaction of the TGF-β/Smad signaling pathway and HIF-1α regulates the expression of PKM2 and PKM1 under hypoxia by promoting c-Myc expression

**Fig. 3:**
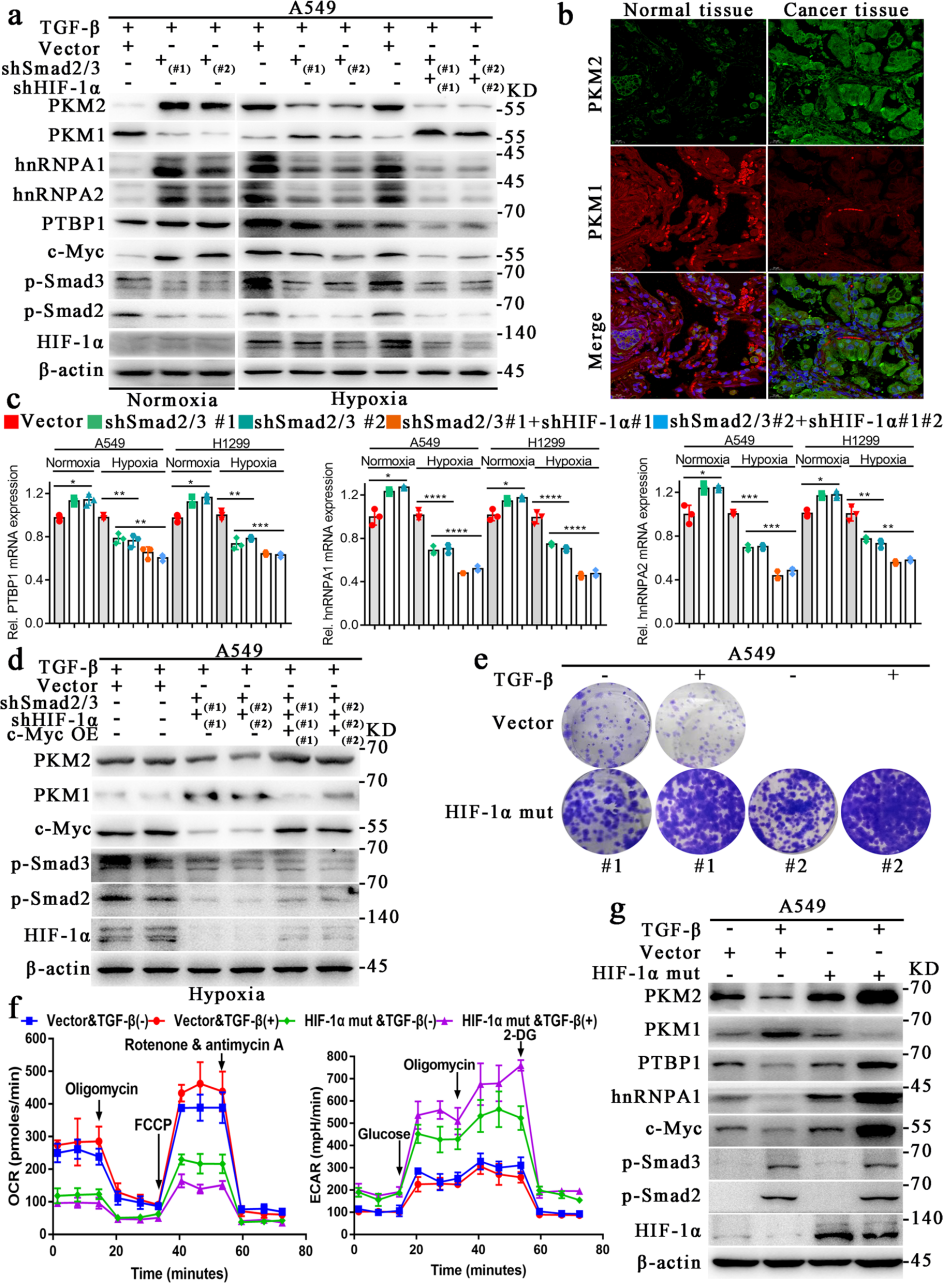
Interaction of the TGF–β/SMAD signaling pathway and HIF-1α regulates the expression of PKM2 and PKM1 under hypoxia by promoting c-Myc expression.** a**, Changes in protein expression of c-Myc and its downstream genes in A549 cells with or without Smad2/3 and HIF-1α knockdown under normoxia or hypoxia. **b**, PKM1 and PKM2 coexisted in cancer cells. PKM2 (Green) was highly expressed in lung adenocarcinoma tissues, while PKM1 (Red) expression was relatively low. The expression of PKM1/2 in adjacent tissues was opposite. **c**, Changes in mRNA expression of downstream genes of c-Myc in A549 and H1299 cells (Treated with TGF-β) with or without Smad2/3 and HIF-1α knockdown under normoxia or hypoxia. **d**, Western blot analysis showing that decreased protein expression of PKM2 and increased PKM1 caused by knockdown of Smad2/3 and HIF-1α can be reversed by overexpression of c-Myc. **e**, Under normoxia, the non-degradable HIF-1α mutants and TGF-β synergistically promoted glycolysis and proliferation of NSCLC cell lines. **f**, ECAR and OCR reflect the effects of the non-degradable HIF-1α mutants and TGF-β on metabolic reprogramming, as examined by Seahorse Extracellular Flux Analyzer XFe96 assay. **g**, Western blot results showed that the non-degradable HIF-1α mutants and TGF-β could synergistically promote the alternative splicing of PKM and significantly increased the ratio of PKM2/PKM1 under normoxia. The concentration of all TGF-β used in this part of the experiment was 5 ng/ml

c-Myc maintains a high PKM2/PKM1 ratio in cells by upregulating PTBP1, hnRNPA1, and hnRNPA2B1 (David et al. [32]). Our study found that the expression of c-Myc was significantly decreased in hypoxia with knockdown of Smad2/3 (Fig. 3a, Supplementary Fig. 3a, b). Therefore, we speculated that TGF-β might affect the expression ratio of PKM2/PKM1 by regulating the expression of c-Myc. Indeed, our results showed that the expression of c-Myc, PTBP1, hnRNPA1, hnRNPA2 (hnRNPA2B1), and PKM1 was increased under normoxia, but the expression of PKM2 decreased in cells with Smad2/3 knockdown. By contrast, these genes’ mRNA and protein expression levels were significantly reduced in A549 and H1299 cells with Smad2/3 knockdown under hypoxia (Fig. 3a, c, Supplementary Fig. 3a). After further blocking the expression of HIF-1α, the expression of these genes was almost completely lost (Fig. 3a, c Supplementary Fig. 3a). These results indicate that TGF-β can inhibit c-Myc, PTBP1, hnRNPA1, hnRNPA2B1, and PKM1under normoxia while promoting the expression of these genes when cultured in hypoxic conditions. These results indicated that TGF-β cross-talk with HIF-1α jointly promotes the increase of the expression ratio of PKM2/PKM1, and ultimately promotes glycolysis of tumor cells. The increased expression ratio of PKM2/PKM1 is an important feature of tumor cells. We selected lung adenocarcinoma tissues that showed high glucose uptake (Supplementary Fig. 3c) by preoperative PET-CT for tissue immunofluorescence staining to verify the expression of PKM1/2 in lung adenocarcinoma. We found that PKM2 was highly expressed in lung adenocarcinoma tissues, while the expression of PKM1 was relatively low. Similarly, PKM1 was highly expressed in adjacent tissues, while PKM2 was low (Fig. 3b, Supplementary Fig. 3d).

We then tried to overexpress c-Myc to determine whether the downregulated PKM2/PKM1 ratio in hypoxia caused by blocking HIF-1α and TGF-β/Smad signaling could be reversed by overexpressing c-Myc. Compared to cells with knockdown of Smad2/3 and HIF-1α only, the PKM2/PKM1 ratio was overturned following overexpression of c-Myc in both A549 and H1299 cells (Fig. 3d, Supplementary Fig. 3e). These results indicate that the downregulating effect of TGF-β on the PKM2/PKM1 ratio under normoxia could be converted in hypoxia by crosstalk with HIF-1α via regulation of the expression of c-Myc.

Hypoxia may elicit additional signaling or metabolic effects that can convolute the interpretation of the data. To further clarify whether HIF-1α mediated the regulatory effect of TGF-β on PKM1/2 alternative splicing under normoxia and hypoxia under hypoxia, we constructed the non-degradable HIF-1α mutants (Supplementary Fig. 3f) to assess whether HIF-1α suffices to drive the TGF-β effects seen in hypoxia under normoxic conditions [33]. We found that the non-degradable HIF-1α mutants and TGF-β synergistically promoted glycolysis and proliferation of cancer cells under normoxic conditions, which was completely consistent with previous results (Fig. 3e, f, g, Supplementary Fig. 3 g).

### Smad3 regulates the expression of c-Myc by forming a complex with HIF-1α to regulate cell cycle-related genes and glycolysis under hypoxia

**Fig. 4:**
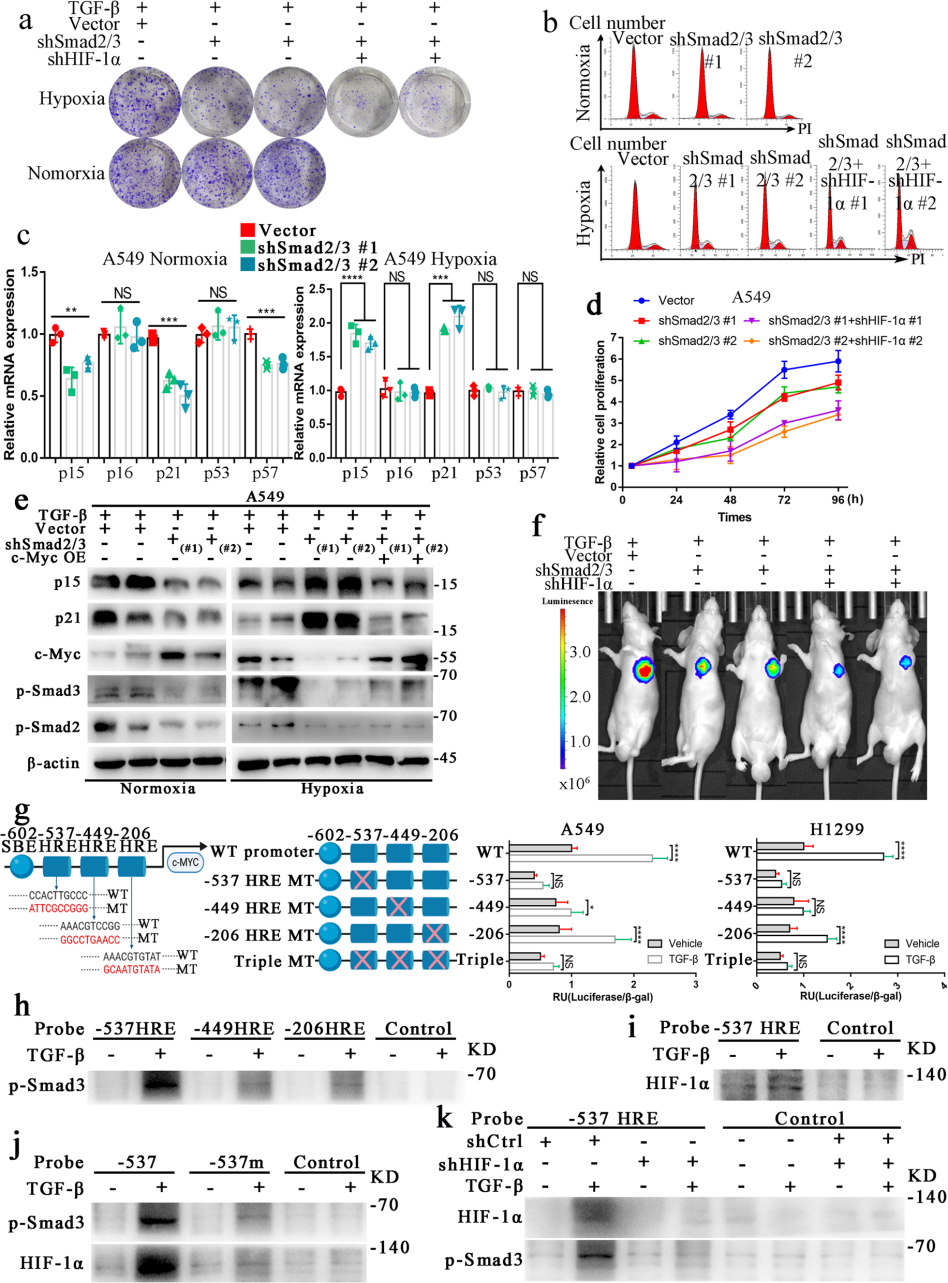
Smad3 regulates the expression of c-Myc by forming a complex with HIF-1α to regulate cell cycle-related genes and glycolysis in hypoxia.** a**, Clone-forming ability of A549 cells was decreased following knockdown of Smad2/3; this ability was further reduced when combined with knockdown of HIF-1α under hypoxia. Under normoxia, the knockdown of Smad2/3 did not have as dramatic of an effect on its clone-forming ability. **b**, Cell cycle assay showed changes in A549 cells with Smad2/3 knockdown; however, a G2/M delay was observed in cells with knockdown of Smad2/3 and HIF-1α. **c**, qRT-PCR analysis of mRNA levels of cell cycle-related genes in A549 cells with the indicated treatment under normoxia or hypoxia. **d**, Depletion of Smad2/3 and HIF-1α significantly attenuated the growth of A549 cells under hypoxia. **e**, Cell cycle-related genes showed contrasting changes in protein expression levels with knockdown of Smad2/3 in normoxia and hypoxia; these effects could be reversed by overexpression of c-Myc. **f**, Changes in the relative fluorescence intensity of subcutaneous tumors with the indicated treatments in NOD/SCID mice. **g**, The c-Myc-Luc structure was mutated at each putative HRE (−537, −449, and –206) or at all three sites and transfected into A549 and H1299 cells. Cells were treated with 5 ng/ml TGF-β or vehicle control for 24 h under hypoxia. Luciferase assay results, corrected by β-galactosidase, are shown as the mean of three separate experiments ± SD analyzed by two-way ANOVA. **h**, pSmad3 binding to DNA precipitation products, especially products pulled down by the −537 HRE probe using DNA affinity precipitation assay (DNPA). **i**, TGF-β promotes HIF-1α binding to the –537 HRE probe of the c-Myc promoter. **j**, DNPA using wild-type or mutant −335 HRE probe with A549 nuclear lysates. **k**, DNPA shows the binding abilities of HIF-1α and p-Smad3 to the –537 HRE probe in A549 cells with or without HIF-1α knockdown. The figures h-k were conducted under hypoxia. The concentration of all TGF-β used in this part of the experiment was 5 ng/ml. All error bars are mean ± SD. NS, not significant. ***P < 0.001, *P < 0.05; determined by two-tailed Student’s *t*-test (95% confidence interval). HRE, HIF-1α response element; WT, wild-type; MT, mutant

c-Myc is an essential gene that regulates the cell cycle and proliferation of tumor cells. To investigate its effect on the cell cycle and proliferation, we assessed the cell cycle and clone formation ability of cells with knockdown of Smad2/3 or/and HIF-1α. The colony formation assay showed that TGF-β inhibits cell proliferation in normoxia, but can promote proliferation under hypoxia (Supplementary Fig. 4e). The clone formation ability of cells in hypoxia was decreased significantly in the absence of HIF-1α and blocking of the TGF-β/Smad signaling pathway while it was significantly increased in normoxia (Fig. 4a). Under the normoxic condition, the proportion of G2/M phase Smad2/3 knockdown cells were decreased, but this proportion increased when cells were cultured in hypoxia. With knockdown of both Smad2/3 and HIF-1α, the proportion of cells in G2/M phase increased further (Fig. 4b, Supplementary Fig. 4c). The expression of p15 and p21 decreased with knockdown of Smad2/3 under normoxia but increased under hypoxia. The increase in p15 and p21 expression caused by knockdown of Smad2/3 under hypoxia was reversed following overexpression of c-Myc (Fig. 4c, e, Supplementary Fig. 4a, b). Moreover, the cell proliferation assay showed that the promoting effect of TGF-β/Smad signaling on cell proliferation was significantly reversed with the knockdown of Smad2/3 in hypoxia. This effect was more robust with the knockdown of both Smad2/3 and HIF-1α (Fig. 4d–f).

To elucidate how TGF-β and HIF-1α regulate the expression of c-Myc, we first predicted the possible binding sites of Smad2, Smad3 (Smad binding elements [SBEs]), and HIF-1α (HBE, also named HIF-1α response elements [HRE]) in the promoter region of c-Myc using the Jaspar database. The top three binding sites of Smad3 on the c-Myc promoter region were -803 SBE, -602 SBE, and -256 SBE. Using a dual-luciferase reporter assay, we found human c-Myc-Luc constructs with mutation of -602 SBE displayed weak activity and no longer responded to TGF-β under hypoxia. However, the other two mutations (-803 and -256 SBE) still retain the response to TGF-β (Supplementary Fig. 4 g-h). Therefore, we confirmed that -602 SBE (5-CCGTCTAGCACCT-3’) was an effective binding site for Smad3. Similarly, we found that -537 HRE (5-CCACTTGCCC-3’) is an effective binding site between HIF-1α and the c-Myc promoter region (Fig. 4 g). A DNA precipitation assay (DNPA) with probes containing each putative HRE and the surrounding sequence was used to precipitate interacting proteins from A549 nuclear lysates treated with 5 ng/ml TGF-β or vehicle control for 8 h under hypoxia. Our results showed that TGF-β could enhance p-Smad3 (Fig. 4 h) and HIF-1α (Fig. 4i) binding to the -537 HRE probe, and this effect was disrupted with a mutant -537 HRE probe (Fig. 4j). With knockdown of Smad3 or HIF-1α, this effect of TGF-β was weakened or blocked (Fig. 4k, Supplementary Fig. 4d). To explore if Smad3 and HIF-1α formed a complex to regulate c-Myc expression cooperatively, we performed a co-immunoprecipitation assay, which showed that p-Smad3 could form a complex with HIF-1α (Supplementary Fig. 4f). Moreover, western blot experiments proved that the expression of c-MYC was highest when both HIF-1α and p-Smad3 were highly expressed (Supplementary Fig. 4i). These results suggest that p-Smad3 regulates the expression of c-Myc by forming a complex with HIF-1α, which could be enhanced by TGF-β, to regulate cell cycle-related genes and glycolysis in hypoxia.

*HIF-1α reverses the inhibitory effect of TGF-β/Smad signaling on c-Myc by binding to the MH2 domain of Smad3 under hypoxia*.

**Fig. 5:**
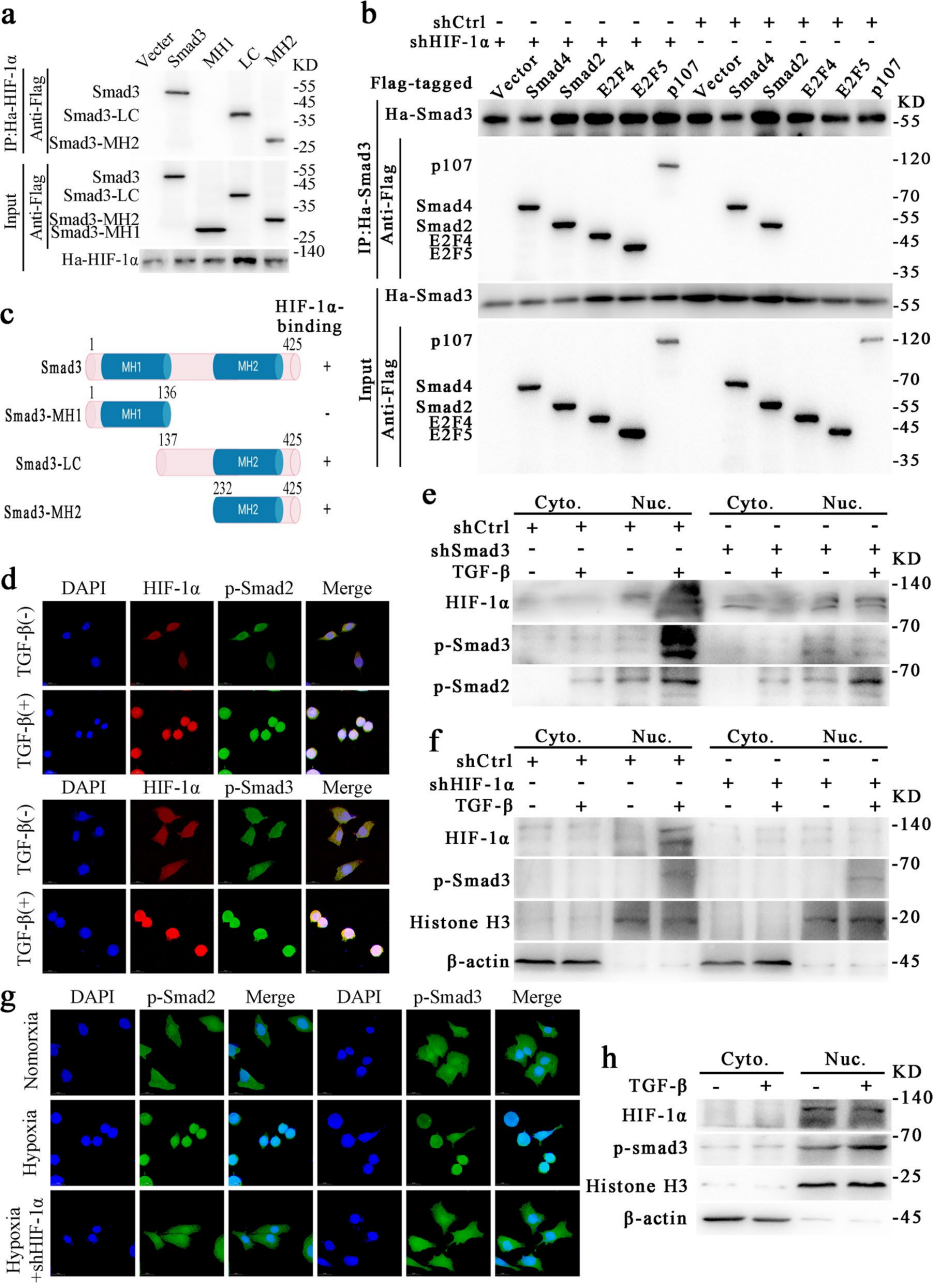
HIF-1α reverses the inhibitory effect of TGF-β/Smad signaling on c-Myc by binding the MH2 domain of Smad3 under hypoxia.** a**, HIF-1α directly binds the MH2 domain of Smad3. Flag-tagged Smad3 deletion mutants were synthesized *in vitro* and HA-HIF-1α bound proteins and input were detected by western blotting. **b**, HIF-1 and p107/E2F4/E2F5 competitively bind the MH2 domain of Smad3. 293T cells were transfected with expression plasmids encoding Flag-tagged genes as indicated as well as HA-Smad3. Co-immunoprecipitation and western blotting were performed. **c**, Schematic diagram of deletion constructs containing different Smad3 domains. Smad3 domains that bind HIF-1α are shown. **d, e**, HIF-1α co-localized with Smad2/3 in the nucleus as shown by western blotting (**e**) and immunofluorescence (**d**). Co-localization was enhanced by TGF-β treatment (scale bars, 20 μm). **f, g**, HIF-1α enhanced p-Smad2 and p-Smad3 nuclear localization, which was suppressed by knockdown of HIF-1α, as shown by western blotting (**f**) and immunofluorescence (**g**). **h**, TGF-β promotes the expression of HIF-1α and p-Smad3 in the nucleus. The concentration of all TGF-β used in this part of the experiment was 5 ng/ml

To study the possible binding domains of p-Smad3 and HIF-1α when forming a complex, we synthesized and transfected Flag-tagged Smad3 deletion mutants into 293T cells. Co-immunoprecipitation indicated that HIF-1α could bind p-Smad3 at the MH2 domain of p-Smad3 (Fig. 5a, c). It has been shown that Smad3 forms a Smad3-p107-E2F4/5 transcription factor complex when cultured in normoxic conditions, thus inhibiting the expression of c-Myc (Chen et al. [3]). These results were consistent with our experimental results described above (Fig. 3a, b). However, this inhibition was abolished under hypoxia. We constructed expression plasmids encoding Flag-tagged E2F4, E2F5, Smad2, Smad4, and p107, and HA-tagged Smad3. These plasmids were transferred into 293T cells with or without HIF-1α knockdown and cultured under hypoxia for 24 h before protein extraction. Immunoprecipitation showed that Smad3 could not bind E2F4, E2F5, and p107 under hypoxia but could bind these proteins after HIF-1α knockdown (Fig. 5b). These results suggest that HIF-1α, which is upregulated under hypoxia, affected E2F4, E2F5, p107, and Smad3 to form a c-Myc transcription regulatory complex, thus relieving the inhibition of c-Myc by TGF-β/Smad signaling.

We showed that TGF-β could promote the nuclear translocation of HIF-1α, p-Smad2, and p-Smad3 by immunofluorescence assay and western blotting; this translocation was blocked by knockdown of Smad3 (Fig. 5d, e, and h). Also, hypoxia-induced HIF-1α promoted the nuclear translocation of p-Smad2 and p-Smad3; this effect was blocked by the knockdown of HIF-1α (Fig. 5 g–f). These results suggest that TGF-β enhanced p-Smad3 and HIF-1α binding to the c-Myc promoter by promoting the nuclear translocation of HIF-1α and p-Smad3.

### TGF-β significantly promotes tumorigenesis under hypoxia in vivo, which can be blocked by knockdown of Smad2/3 and/or HIF-1α

**Fig. 6:**
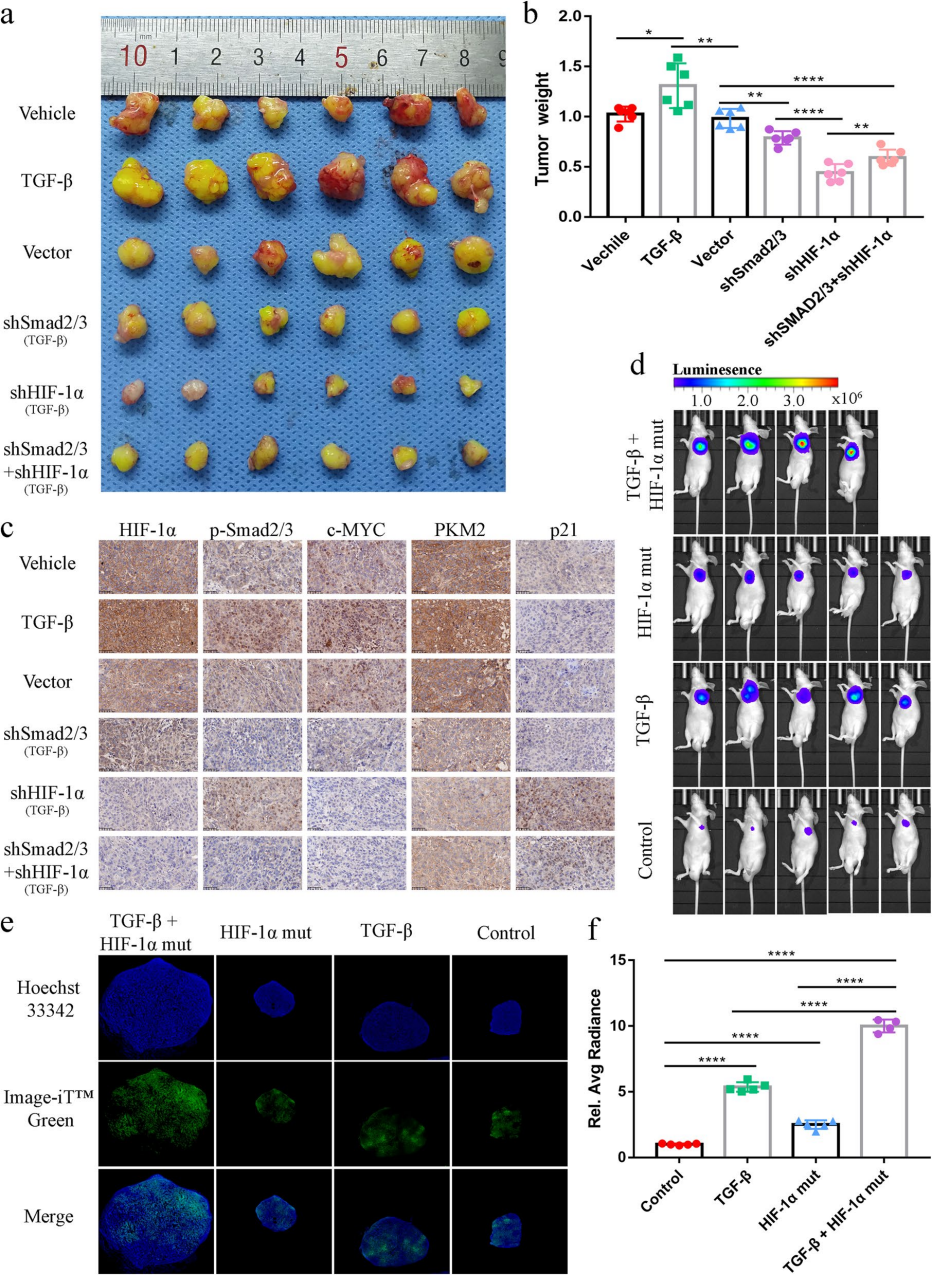
TGF-β can significantly promote tumorigenesis under hypoxia ***in vivo***, which can be blocked by knockdown of Smad2/3 and HIF-1α. **a**, Compared with Smad2/3 knockdown or both Smad2/3 and HIF-1α knockdown, HIF-1α knockdown alone can more effectively inhibit TGF-β-driven tumor formation. A549 cells stably expressing GFP were injected subcutaneously into the armpit of the forelimb of nude mice. TGF-β (50 µl, 5 ng/ml) was injected into a parallel subcutaneous tumor group once a week. Actual tumors are shown. **b**, Statistical analysis of tumor formation in **a**, n = 6 mice/group. **c**, Immunohistochemical staining of paraffin sections prepared from subcutaneous tumors of nude mice (scale bars, 50 μm). TGF-β plays opposing roles in regulating PKM2 and p21 expression by enhancing the crosstalk of p-Smad2/3 and HIF-1α. **d**, Changes in the relative fluorescence intensity of subcutaneous tumors with the indicated treatments in NOD/SCID mice (5 mice in each group, one of them died in the group of TGF-β + HIF-1α mut). **e**, A549 subcutaneous tumor tissue fluorescence showed that Image-iT™ Green Hypoxia Reagent detected some hypoxic areas. **f**, Taking the blank control group as a reference, the results showed that the fluorescence intensity of the subcutaneous tumors of nude mice was significantly higher with indicated treatments. HIF-1α mut +TGF-β group had the strongest fluorescence intensity. ****P < 0.0001

To determine the effect of TGF-β under hypoxia on tumors *in vivo*, we subcutaneously injected A549 cells with or without Smad2/3 knockdown and/or HIF-1α knockdown into nude mice. We then compared their proliferation potential *in vivo*. TGF-β significantly promoted tumor formation, blocking Smad2/3 and/or HIF-1α could significantly decrease tumor size; the greatest inhibitory effect on tumors was observed with knockdown of only HIF-1α (Fig. 6a, b, Supplementary Fig. 5a). These results suggest that blocking the expression of HIF-1α may partially restore the proliferation inhibitory effect of TGF-β/Smad signaling. Immunohistochemistry showed that TGF-β could promote c-Myc and PKM2 and inhibit the expression of p21 *in vivo*. The expression of c-Myc and PKM2 decreased, but the expression of p21 increased with knockdown of Smad2/3 and/or HIF-1α, especially with knockdown of only HIF-1α (Fig. 6c). These results are consistent with the *in vitro* results.

To further explore the effects of HIF-1α and TGF-β on the tumorigenesis of non-small cell lung cancer cells in vivo, we injected A549 cells transfected with luciferase plasmid into nude mice to observe the effects of different treatments on their tumorigenesis ability. We found that the non-degradable HIF-1α mutants and TGF-β synergistically promoted tumor formation significantly (Fig. 6d, f). These results were consistent with the experimental results obtained in vitro. In addition, to verify whether our subcutaneous tumor model was suitable for simulating the hypoxia state of tumor in vivo, we verified whether the tumor cells of subcutaneous tumor were in a hypoxic environment with Image-iT™ Green Hypoxia Reagent. We found that the A549 subcutaneous tumor did have hypoxic areas (Although not all areas showed hypoxia) (Fig. 6e). These results indicated that our model could indeed simulate the hypoxia environment of the tumor in vivo.

### High expression of HIF-1α is associated with increased expression of c-Myc and PKM2 but decreased expression of p21 in NSCLC patients

**Fig. 7:**
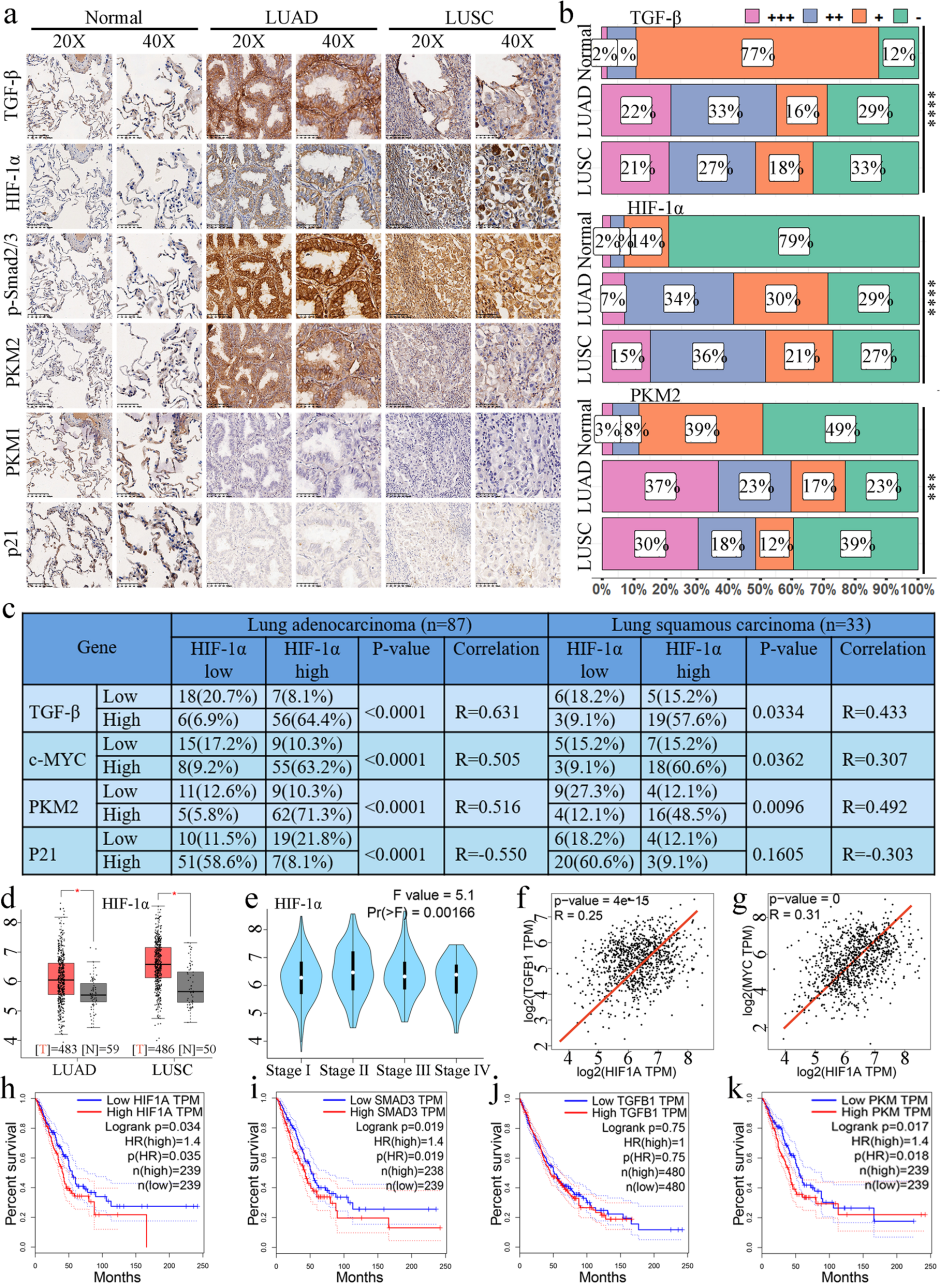
High expression of HIF-1α is associated with high expression of c-Myc and PKM2 but low expression of p21 in NSCLC patients.** a**, Representative IHC images of TGF-β, HIF-1α, p-Smad2/3, PKM1/2, and p21 staining in lung adenocarcinoma and lung squamous cell carcinoma compared with normal lung tissue. Scale bars, 50 μm. **b**, Expression intensity distribution of TGF-β, HIF-1α, and PKM2 based on IHC staining in lung adenocarcinoma, squamous cell carcinoma, and adjacent tissues. IHC scores were divided into four levels according to IHC staining analyzed by ImageJ software. ****P < 0.0001; determined by the two-tailed Student’s *t*-test (95% confidence interval). **c**, Relationship between the expression of HIF-1α and TGF-β, c-Myc, PKM2, and p21 in lung adenocarcinoma and squamous cell carcinoma. The table shows the number of cases, the percentage of positive staining cases in the corresponding group, and the statistical significance based on the Student’s *t*-test and Pearson correlation analysis. **d, e**, Based on TCGA data analysis, the expression of HIF-1α in lung adenocarcinoma and squamous cell carcinoma was significantly higher than that in normal tissues (**d**) (*P < 0.05, by two-tailed *t*-test). HIF-1α also increased significantly with the development of lung cancer (**e**). **f, g**, Based on TCGA data analysis, the expression of HIF-1α was positively correlated with the expression of TGF-β (**f**) and c-Myc (**g**). **h–k**, Kaplan–Meier survival analysis revealed that the prognosis of patients with high expression of HIF-1α (**h**), Smad3 (**i**), and PKM (**k**) is worse; this was not reflected in patients with higher expression of TGF-β (**j**)

**Fig. 8:**
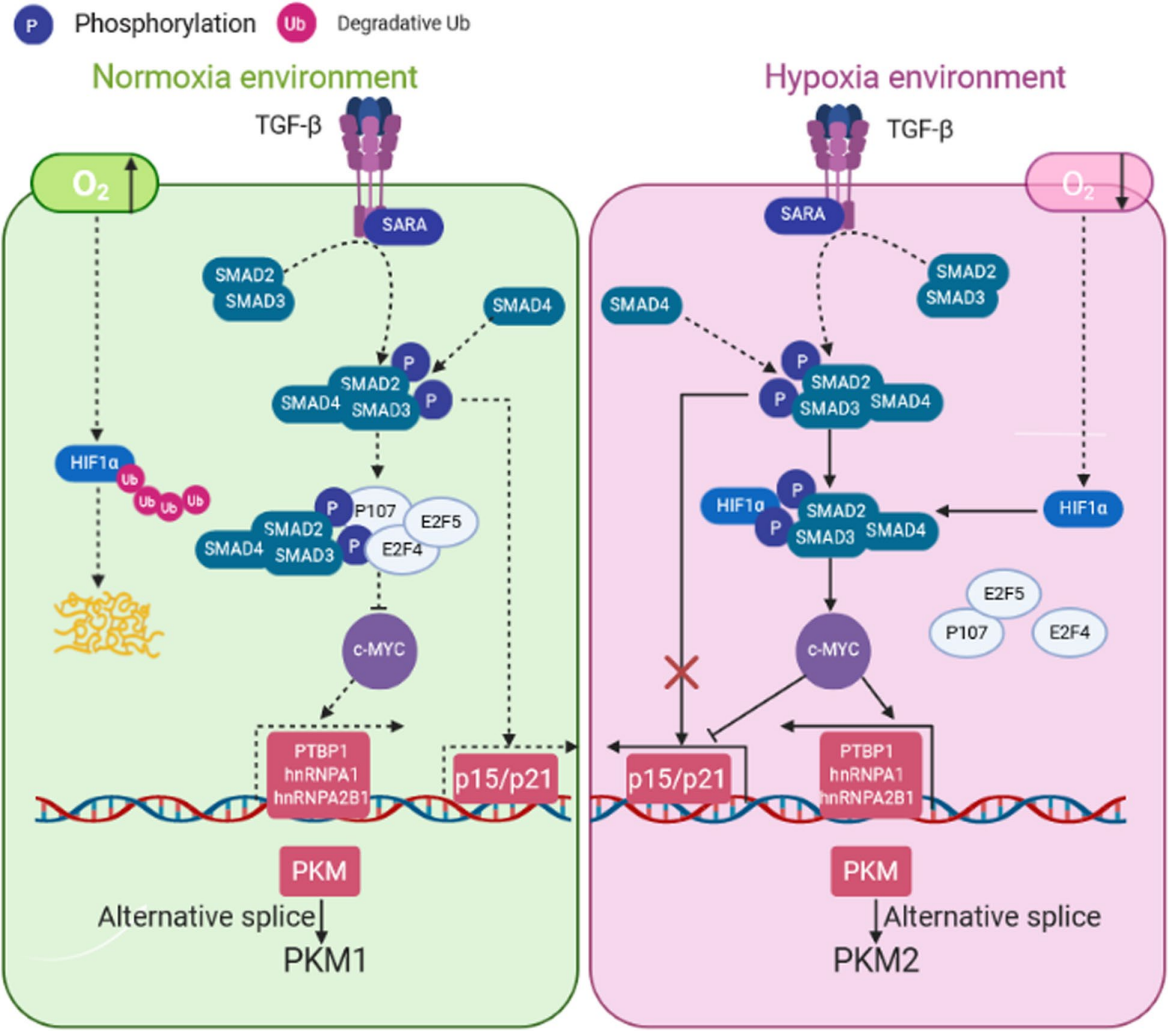
Diagram of the TGF-β functional switch driven by increased HIF-1α under hypoxia by changing Smad partners in cancer cells. HIF-1α can be ubiquitinated and rapidly degraded under normoxic conditions. TGF-β can inhibit cell proliferation by promoting p15/p21 expression through the Smad transcription factor complex. On the other hand, Smads can bind p107 and E2F4/5 to inhibit the expression of c-Myc, thus regulating the variable splicing of PKM precursor RNA. Overall, TGF-β can inhibit cell proliferation by regulating the cell cycle and reducing glycolysis under normoxia (left). HIF-1α protein is stable in the cell under hypoxia. TGF-β promotes nuclear translocation of HIF-1α and enhances its binding with p-Smad3. High expression of HIF-1α competitively binds the MH2 domain of p-Smad3, thus relieving the inhibitory effect of the Smad-p107-E2F4/5 complex on c-Myc. c-Myc inhibits the expression of p15/p21 and regulates the variable splicing of PKM precursor RNA to express more PKM2 to promote glycolysis (right). The solid line represents the new findings described in this study, and the dotted line represents the results of previous studies

Our results thus far suggested that the tumor suppressor function of TGF-β in normoxia is by reducing c-Myc and the PKM2/PKM1 ratio. In contrast, the proliferation-promoting function of TGF-β is switched on by interacting with HIF-1α under hypoxia in NSCLC. To validate our findings and determine the clinical correlation, we detected the expression of members of the TGF-β signaling pathway (p-Smads, HIF-1α, c-Myc, PKM2, and PKM1) and their relevance to TGF-β in patient-derived tissue chips. High expression of TGF-β, HIF-1α, and PKM2 was found in most patients (Fig. 7a, b). The expression of p21 and PKM1 was low in most patients with lung adenocarcinoma (LUAD) or lung squamous cell carcinoma (LUSC) (Fig. 6a, Supplementary Fig. 5a). Notably, the high expression of HIF-1α was significantly associated with increased c-Myc and PKM2 but negatively correlated with the expression of p21 (Fig. 7c). Together, the data of our human tissue microarray supported the conclusions of our in vitro studies. Namely, HIF-1α crosstalk with TGF-β signaling to promote tumor progression by increased expression of c-Myc and PKM2 under hypoxia through changing Smad partners in cancer cells (Fig. 8).

We next analyzed the public database to explore further the correlation between gene expression and the impact on the clinical prognosis of patients. The TCGA data showed that HIF-1α was significantly increased in LUAD and LUSC, and the expression of HIF-1α was higher in late-stage than in early-stage patients (Fig. 7d, e). We also found significant expression correlations among TGF-β, HIF-1α, Smad2, Smad3, c-Myc, and p21 (Fig. 7f, g, Supplementary Fig. 6 g–k). Kaplan–Meier survival analysis showed that patients with high expression of HIF-1α, Smad2, Smad3, PKM, and PTBP1 had significantly lower overall survival (Fig. 7 h–k, Supplementary Fig. 6b–f).

## Discussion

Cancer cells show a distinct metabolic phenotype, characterized by increased glycolysis, which produces lactate rather than undergoing mitochondrial oxidative phosphorylation. This phenomenon is called the Warburg effect or aerobic glycolysis [34]. Metabolic reprogramming of cancer cells supports the biomass production required for rapid proliferation. The selective expression of an isoform of the glycolytic enzyme pyruvate kinase M (PKM) is related to the metabolic phenotype of cancer cells [34]. The reversal of PKM1 to PKM2 is observed in most cancers, which partly explains the Warburg effect observed in cancer cells and ensures the highest incidence of tumors [21, 22, 32]. The alternative splicing of PKM is dependent on splicing factors hnRNPA1, hnRNPA2, and PTB, which attach to the exon 9 (E9) 5’ splice site (EI9) to block E9 inclusion in the transcript [21, 22, 32]. A genome-wide chromatin immunoprecipitation (ChIP) assay showed that c-Myc could bind to the promoter regions of PTBP1, hnRNPA1, and hnRNPA2B1 and upregulate their expression levels [32]. Here, we found that TGF-β could significantly increase glucose uptake and lactate production by increasing the expression of PKM2 under hypoxia. Simultaneously, TGF-β can also considerably promote the expression of several other vital glycolysis-related genes under hypoxic conditions, such as LDHA, PFKP, and HK2.

Interestingly, TGF-β significantly inhibited glycolysis under normoxia. However, TGF-β facilitated tumor cell glycolysis in hypoxia. The effect of TGF-β on the metabolic reprogramming in hypoxia was caused by the release of the inhibition of c-Myc. As mentioned above, c-Myc is a vital regulator of genes such as hnRNPA1, hnRNPA2B1, and PTBP1. Studies have shown that these genes can regulate the alternative splicing of several glycolysis-related genes to regulate the glucose metabolism of tumor cells [18, 32]. Our results confirmed that the function of TGF-β in tumor cells could switch in normoxic and hypoxic environments. We found that the expression of HIF-1α was significantly different under normoxia and hypoxia, consistent with previous results [35]. The present study confirms that HIF-1α plays an essential role in the functional transformation of TGF-β in cancer cells.

HIF-1α acts as a regulator of intermediary metabolism in mammalian development and adult physiology, especially in the control of glycolysis under low O_2_ levels, and as an activator of genes involved in glucose metabolism regulation [36]. It has been suggested that the expression of HIF-1α is decreased in the early stage of tumors compared with the advanced stage [37, 38]. With an increase in tumor size, the expression of HIF-1α increases as the level of hypoxia increases, which will further promote tumor development. Previous studies have shown that patients with increased expression of HIF-1α have a significantly worse prognosis [38, 39]. Our study also similarly indicated that high expression of HIF-1α was related to significantly shorter overall and disease-free survival time (P = 0.003 and P = 0.001, respectively; Cox regression analysis). Some transcription factors have different regulatory effects on downstream genes under hypoxia and normoxia, which is thought to be related to HIF-1α [40]. HIF-1α can regulate glucose metabolism and tumor cell proliferation by regulating other transcription factors [41]. HIF-1α can also regulate the expression of many glycolysis-related genes and promote tumor angiogenesis [42]. In this study, we found that HIF-1α could change the regulatory effect of TGF-β on glucose metabolism and the cell cycle by binding Smad3, a key molecule in the TGF-β signaling pathway. HIF-1α enabled the dual roles of TGF-β in regulating glucose metabolism and the cell cycle of tumor cells under normoxia and hypoxia.

Many studies have shown that TGF-β plays an inhibitory role in the early stage of tumors and promotes tumor progression in advanced cancer [2]. Many genes may play an essential role in this transformation, including PSPC1, 14-3-3ζ, and SOX4 [6, 10, 43]. Here, we found that HIF-1α also played a similar role. The stability of HIF-1α is different under normoxia and hypoxia [41]. HIF-1α can be degraded rapidly under normoxia but can stably exist under hypoxia [44]. This characteristic of HIF-1α enabled TGF-β to inhibit tumor growth in the early stage of tumors, while HIF-1α protein was highly expressed in advanced solid tumors due to hypoxia, thus reversing the inhibitory effect of TGF-β on glycolysis and the cell cycle of tumor cells. The increased expression of HIF-1α changed Smad3 binding partners by binding Smad3. The change in Smad3 partners affected its regulation of downstream genes, which prompted TGF-β to play an opposite role in regulating glucose metabolism of tumor cells in normoxia and hypoxia. HIF-1α and Smad3 have two binding sites close to each other on the c-Myc promoter. The formation of the HIF-1α-Smad3 transcriptional complex significantly promoted the expression of c-Myc. Our results showed that it was the formation of this complex that led to the dual regulation of TGF-β on glucose metabolism and the cell cycle of tumor cells under normoxia and hypoxia. This result is also consistent with the previously observed phenomenon that TGF-β inhibits tumor growth in the early stages but promotes tumor progression in advanced stages [2].

The E2F proteins play an essential role in the regulation of c-Myc by TGF-β. These proteins rely on a conserved DNA binding domain to bind the target gene promoter. As transcription factors, E2Fs bind the 5’-TTTCCCGC-3’ (or slight variations of this sequence) consensus binding site in the target promoter sequence [45]. E2F1–E2F5 contains a transactivation domain that mediates the recruitment of transcriptional co-activators or one of three pocket proteins, termed Rb, p107, and p130, to suppress E2F activity by masking their transactivation domains [46, 47]. Members of the E2F family are thought to contain transcriptional activators and inhibitors. Studies have found that E2F1, E2F2, and E2F3a are mature transcriptional activators and can drive gene expression programs, determine cell cycle commitment, orderly transition from G1 to S phase, and promote cell proliferation [47, 48]. E2F3b and E2F4–E2F6 belong to the “repressive” E2F branch because they inhibit the transcription of E2F target genes in the quiescent (G0) and early G1 phases (when associated with pocket proteins) [47]. A previous study found evidence that the Smad-p107-E2F4/5 complex is essential in the response of c-Myc to TGF-β [3]. In the present study, we found that increased expression of HIF-1α affected the binding of Smad3 to E2F4, E2F5, and p107 in a hypoxic environment, thereby affecting the formation of the Smad-E2F4/5-p107 transcription complex because HIF-1α can also bind the MH2 domain of Smad3. Previous studies confirmed that E2F4/5 and p107 could bind the MH2 domain of Smad3  [3]. The change in the partner of the transcription factor can alter its regulatory effects on target genes [6, 10, 43]. We speculate that when HIF-1α binds the MH2 domain of Smad3, it may affect the binding of E2F4/5 and p107 to DNA due to space occupation. The formation of the HIF-1α-Smad3 complex transformed the TGF-β/Smad signaling pathway, which originally inhibited c-Myc, to promote c-Myc expression. The increased expression of c-Myc can regulate glycolysis and the cell cycle by promoting alternative splicing of PKM and inhibiting the expression of p15/p21.

## Conclusions

Our findings indicate that the regulatory role of TGF-β in NSCLC was affected by hypoxic conditions. In the early stage of solid tumors, tumor size is small and the blood supply for tumor cells is relatively rich, so that the oxygen content of tumor cells is relatively high. With increased tumor volume, cells in the middle of the tumor experience hypoxia, and the expression of HIF-1α is significantly increased. HIF-1α can bind Smad3 to form a transcription complex, which changed the partners of Smad3 and caused TGF-β to lose its inhibitory effect on c-Myc. The formation of the HIF-1α-Smad3 complex also caused the TGF-β signaling pathway to losing its regulatory effect on p15/p21. We also found that TGF-β could promote nuclear translocation of Smad3 and HIF-1α and promote their binding to the promoter site of c-Myc. These new findings suggest that HIF-1α switches the inhibitory function of TGF-β to promote NSCLC by forming the Smad-HIF-1α complex.

## Supplementary Information

**Additional file 1: Supplementary Fig. 1.** a, The regulatory network of glycolysis related transcription factors and their target genes in non-small cell lung cancer. The red font represented the transcription factor, the blue font represented the target gene, the purple font represented both the transcription factor and the target gene, and the genes circled in the green box were some of the genes involved in this study. b, Effects of TGF-β on lactate production in A549 and H1299 cells. d, 18 F-FDG microPET imaging of subcutaneously implanted tumor model mice. Xenograft imaging of A549 cells with knockdown of HIF-1α and control groups treated with TGF-β (5 ng/ml, injection once a week, Three nude mice per group). e, g, Effects of TGF-β on glucose uptake in A549 and H1299 cells. f, Heatmap showing the expression of key glycolytic enzymes detected by RT-qPCR after RNA extraction from subcutaneous tumor tissue. ***P < 0.001, **P < 0.01; P-values were calculated with a two-tailed t-test.

**Additional file 2: Supplementary Fig. 2.** a, b, c, mRNA expression of Smad2, Smad3, and PKM was significantly decreased in A549 and H1299 cells following transfection with corresponding shRNAs. d, Western blot analysis showed that total levels of Smad2 and Smad3, as well as those of p-Smad2/3, were decreased. e-f, Under hypoxic conditions, knocking down smad2/3 could reduce the glucose uptake and lactate production of A549 (e,f) and H1299 (g,h) cells, and this effect was more pronounced when simultaneously knocked down HIF-1α.

**Additional file 3: Supplementary Fig. 3.** a, Changes in protein expression of c-Myc and its downstream genes in H1299 cells with or without Smad2/3 and HIF-1α knockdown under normoxia or hypoxia. b, Changes in mRNA expression of c-Myc in A549 and H1299 cells with or without Smad2/3 and HIF-1α knockdown under normoxia or hypoxia. c, The PET-CT images we used to perform tissue immunofluorescence showed high glucose uptake. d, Tissue immunofluorescence experiments confirmed that PKM1/2 coexist in lung adenocarcinoma cells, and the expression of PKM2 (Green) was much higher than that of PKM1 (Red). The expression of PKM1/2 in adjacent tissues was opposite. e, Western blot analysis showing that decreased protein expression of PKM2 and increased PKM1 caused by knockdown of Smad2/3 and HIF-1α can be reversed by overexpression of c-Myc in H1299 cells. f, The pattern diagram showed the specific mutation sites of the non-degradable HIF-1α mutants. g, Cell proliferation experiments showed that the promotion of cell proliferation by TGF-β and the non-degradable HIF-1α mutants was significantly reduced after knocking down PKM2.

**Additional file 4: Supplementary Fig. 4.** a, b, qRT-PCR analysis of mRNA levels of cell cycle-related genes in H1299 cells with the indicated treatment under normoxia or hypoxia. c, Percentage of G1/S/G2 phase A549 cells subjected to the indicated treatments. d, DNPA shows TGF-β enhancing HIF-1α binding to the –537 HRE probe by p-Smad3. e, TGF-β plays a contradictory effect in the clone-forming ability of A549 cells under normoxia and hypoxia. f, p-Smad3 interacts with HIF-1α in A549 and H1299 cells. Cell lysates were collected and immunoprecipitated with the indicated antibodies. g, h, Dual-luciferase reporter assay revealed that the -602 SBE in the promoter of c-Myc was the effective binding site of Smad3 in A549 (g) and H1299 (h) cells. i, Western blot experiments proved that the expression of c-MYC was highest when both HIF-1α and p-Smad3 were highly expressed.

**Additional file 5: Supplementary Fig. 5.** a, Demonstration of tumor-forming ability of A549 cells following different treatments in nude mice. b, Hematoxylin and eosin staining of paraffin sections derived from subcutaneous tumors formed by A549 cells subjected to the indicated treatments in nude mice.

**Additional file 6: Supplementary Fig. 6.** a, Expression intensity distribution of p21 based on IHC staining in lung adenocarcinoma, squamous cell carcinoma, and adjacent tissues. b-f, Based on TCGA data analysis, Kaplan–Meier survival analysis revealed that the prognosis of patients with high expression of CDKN1A (p21) (h) and PTBP1 (f) is worse; this was not demonstrated with high expression of Smad2 (b), c-Myc (c), and HNRNPA1 (e). g–k, Based on TCGA data analysis, expression of HIF-1α was positively correlated with the expression of Smad2 (h) and Smad3 (g), and expression of TGF-β was positively correlated with expression of c-Myc (i) and PKM (j) but negatively associated with CDKN1A (p21) (k).

**Additional file 7.**

## Data Availability

The materials can be applied to the corresponding author for use.

## Abbreviations

TGF-β: transforming growth factor -β
HIF-1α: hypoxia-inducible factor -1α
NSCLC: non-small cell lung cancer
PK: Pyruvate kinase
TCGA: The Cancer Genome Atlas database
SUVmax: the maximum standardized uptake value
ECAR: the extracellular acidification rate
OCR: the oxygen consumption rate
SBEs: Smad binding elements
HRE: HIF-1α response elements
DNPA: DNA precipitation assay
LUAD: lung adenocarcinoma
LUSC: lung squamous cell carcinoma
