# Supplementary figures and images for: HIF-1α switches the functionality of TGF-β signaling via changing the partners of smads to drive glucose metabolic reprogramming in non-small cell lung cancer
Source: J Exp Clin Cancer Res. 2021 Dec 20;40:398. doi: 10.1186/s13046-021-02188-y (PMC8690885; doi:10.1186/s13046-021-02188-y)

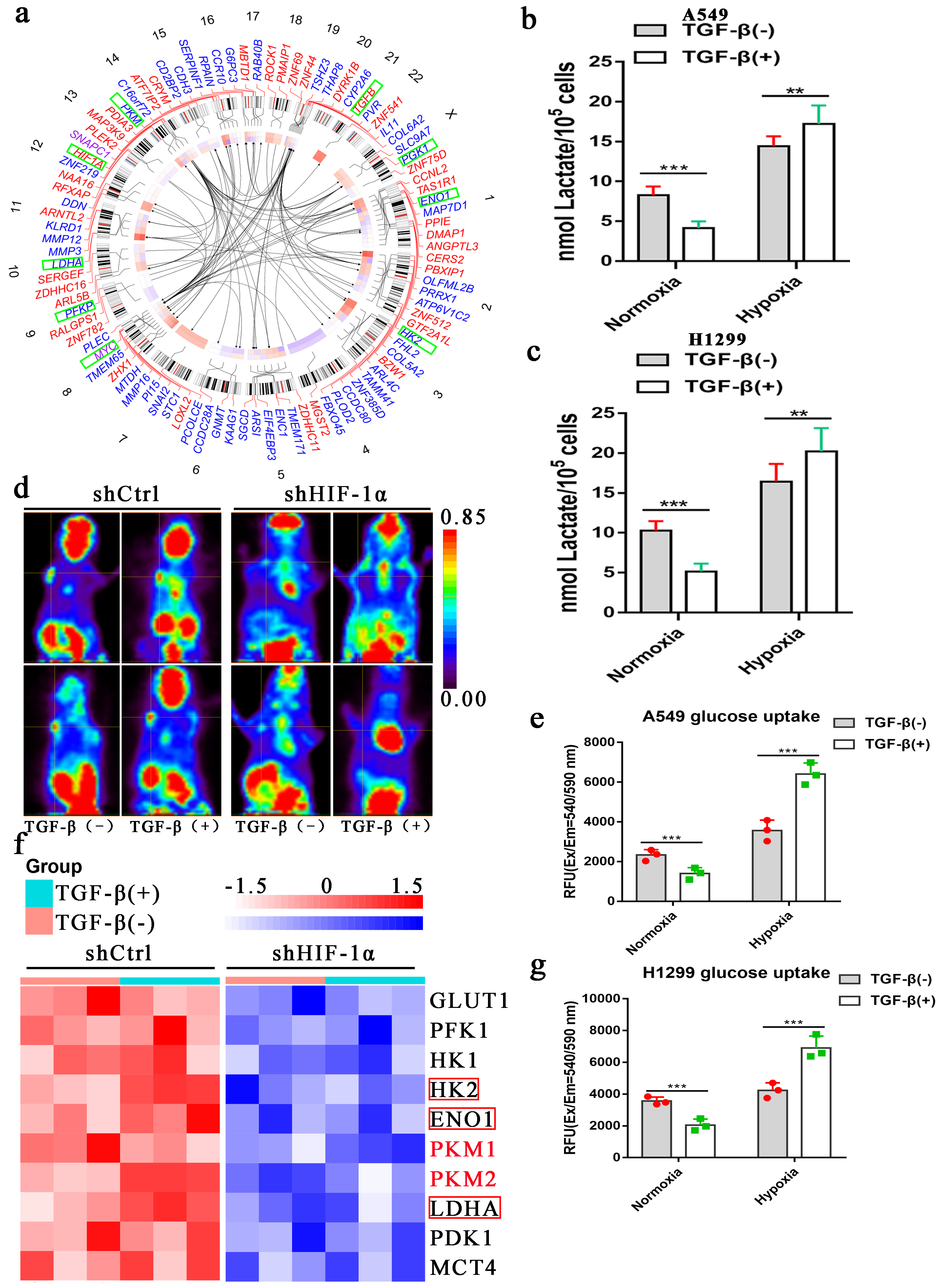

Supplement: Supplementary file 1 — Additional file 1: Supplementary Fig. 1. a, The regulatory network of glycolysis related transcription factors and their target genes in non-small cell lung cancer. The red font represented the transcription factor, the blue font represented the target gene, the purple font represented both the transcription factor and the target gene, and the genes circled in the green box were some of the genes involved in this study. b, Effects of TGF-β on lactate production in A549 and H1299 cells. d, 18 F-FDG microPET imaging of subcutaneously implanted tumor model mice. Xenograft imaging of A549 cells with knockdown of HIF-1α and control groups treated with TGF-β (5 ng/ml, injection once a week, Three nude mice per group). e, g, Effects of TGF-β on glucose uptake in A549 and H1299 cells. f, Heatmap showing the expression of key glycolytic enzymes detected by RT-qPCR after RNA extraction from subcutaneous tumor tissue. ***P < 0.001, **P < 0.01; P-values were calculated with a two-tailed t-test. [file 13046_2021_2188_MOESM1_ESM.tif]

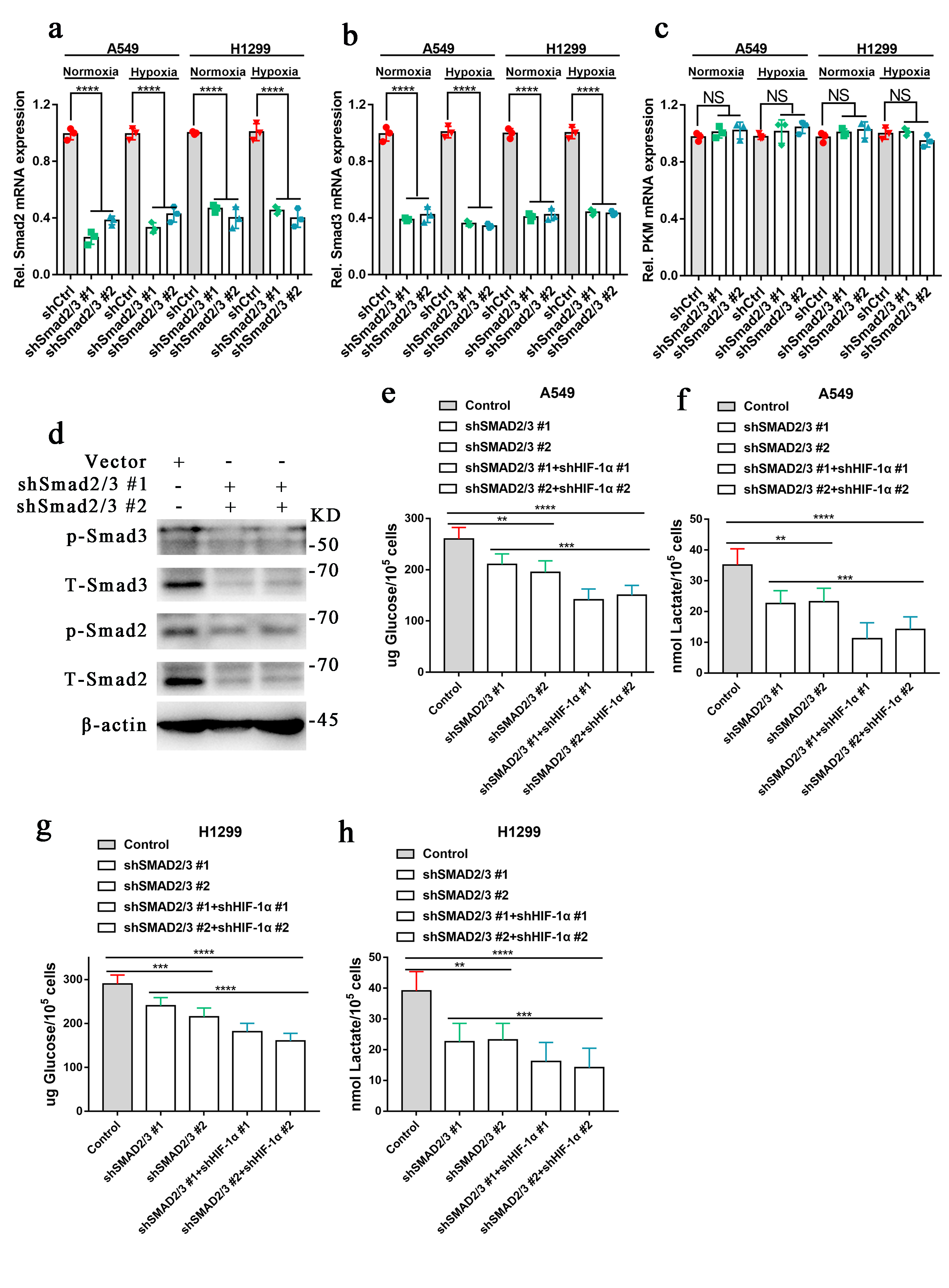

Supplement: Supplementary file 2 — Additional file 2: Supplementary Fig. 2. a, b, c, mRNA expression of Smad2, Smad3, and PKM was significantly decreased in A549 and H1299 cells following transfection with corresponding shRNAs. d, Western blot analysis showed that total levels of Smad2 and Smad3, as well as those of p-Smad2/3, were decreased. e-f, Under hypoxic conditions, knocking down smad2/3 could reduce the glucose uptake and lactate production of A549 (e,f) and H1299 (g,h) cells, and this effect was more pronounced when simultaneously knocked down HIF-1α. [file 13046_2021_2188_MOESM2_ESM.tif]

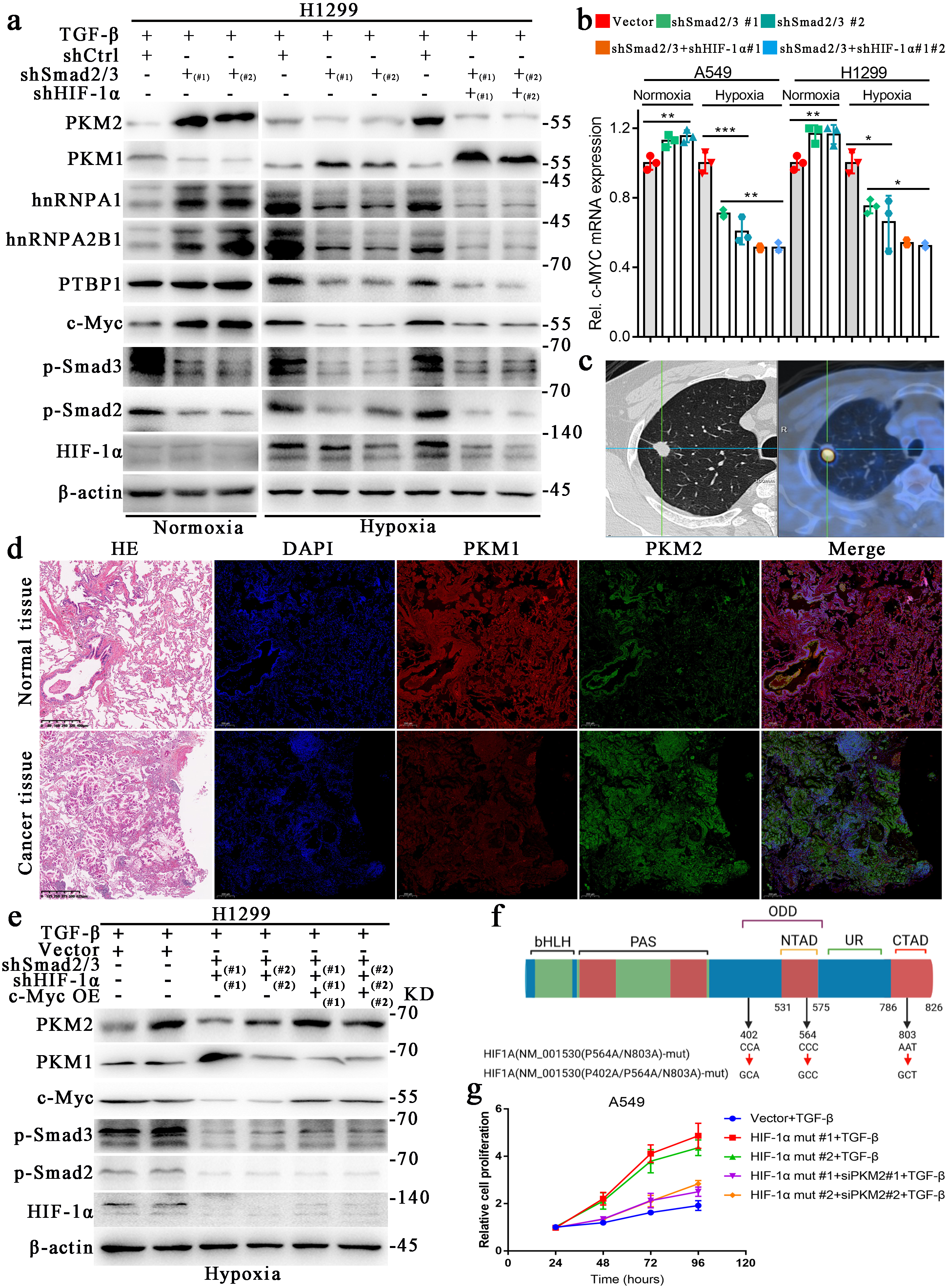

Supplement: Supplementary file 3 — Additional file 3: Supplementary Fig. 3. a, Changes in protein expression of c-Myc and its downstream genes in H1299 cells with or without Smad2/3 and HIF-1α knockdown under normoxia or hypoxia. b, Changes in mRNA expression of c-Myc in A549 and H1299 cells with or without Smad2/3 and HIF-1α knockdown under normoxia or hypoxia. c, The PET-CT images we used to perform tissue immunofluorescence showed high glucose uptake. d, Tissue immunofluorescence experiments confirmed that PKM1/2 coexist in lung adenocarcinoma cells, and the expression of PKM2 (Green) was much higher than that of PKM1 (Red). The expression of PKM1/2 in adjacent tissues was opposite. e, Western blot analysis showing that decreased protein expression of PKM2 and increased PKM1 caused by knockdown of Smad2/3 and HIF-1α can be reversed by overexpression of c-Myc in H1299 cells. f, The pattern diagram showed the specific mutation sites of the non-degradable HIF-1α mutants. g, Cell proliferation experiments showed that the promotion of cell proliferation by TGF-β and the non-degradable HIF-1α mutants was significantly reduced after knocking down PKM2. [file 13046_2021_2188_MOESM3_ESM.tif]

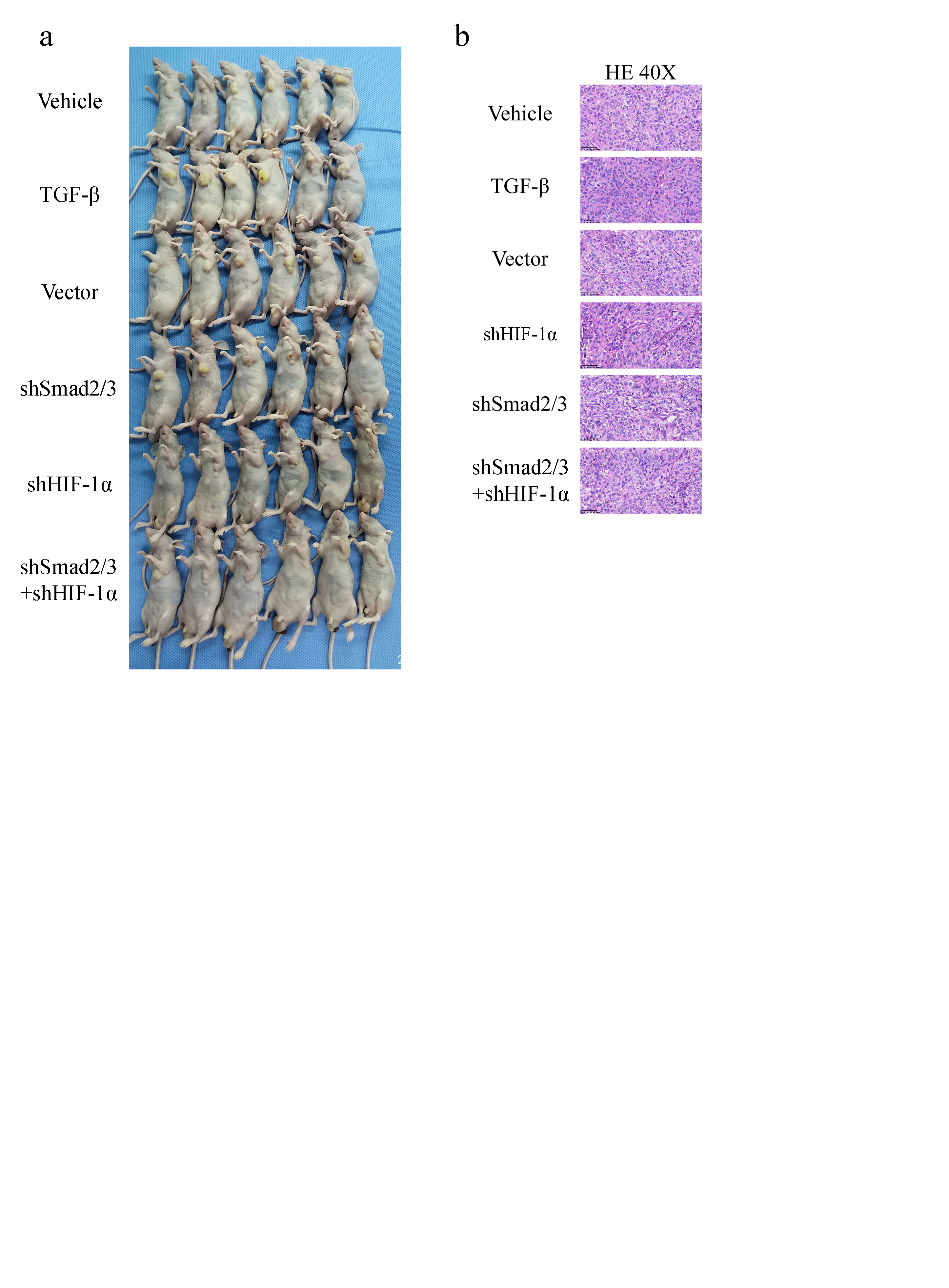

Supplement: Supplementary file 4 — Additional file 4: Supplementary Fig. 4. a, b, qRT-PCR analysis of mRNA levels of cell cycle-related genes in H1299 cells with the indicated treatment under normoxia or hypoxia. c, Percentage of G1/S/G2 phase A549 cells subjected to the indicated treatments. d, DNPA shows TGF-β enhancing HIF-1α binding to the –537 HRE probe by p-Smad3. e, TGF-β plays a contradictory effect in the clone-forming ability of A549 cells under normoxia and hypoxia. f, p-Smad3 interacts with HIF-1α in A549 and H1299 cells. Cell lysates were collected and immunoprecipitated with the indicated antibodies. g, h, Dual-luciferase reporter assay revealed that the -602 SBE in the promoter of c-Myc was the effective binding site of Smad3 in A549 (g) and H1299 (h) cells. i, Western blot experiments proved that the expression of c-MYC was highest when both HIF-1α and p-Smad3 were highly expressed. [file 13046_2021_2188_MOESM4_ESM.tif]

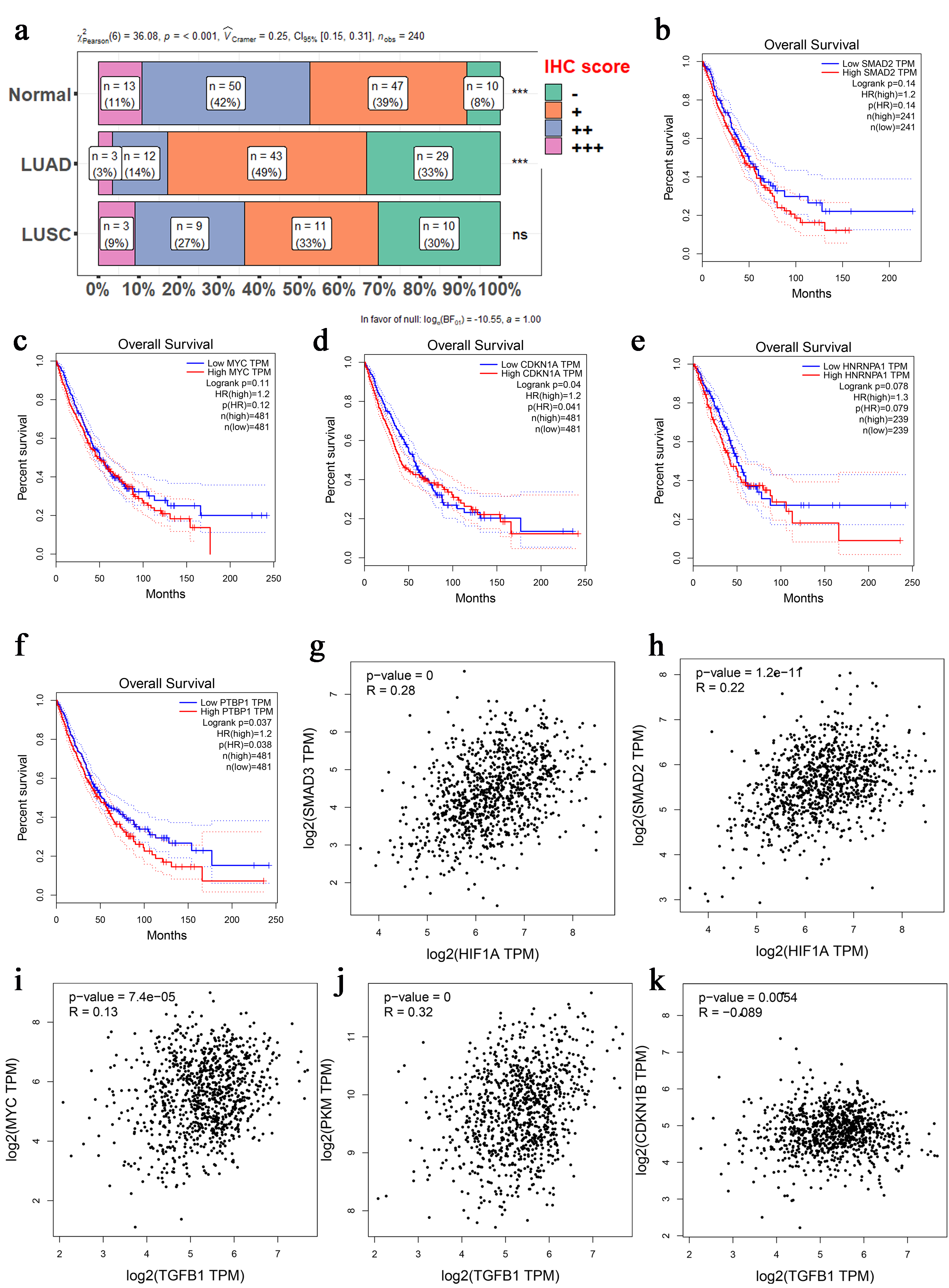

Supplement: Supplementary file 5 — Additional file 5: Supplementary Fig. 5. a, Demonstration of tumor-forming ability of A549 cells following different treatments in nude mice. b, Hematoxylin and eosin staining of paraffin sections derived from subcutaneous tumors formed by A549 cells subjected to the indicated treatments in nude mice. [file 13046_2021_2188_MOESM5_ESM.tif]

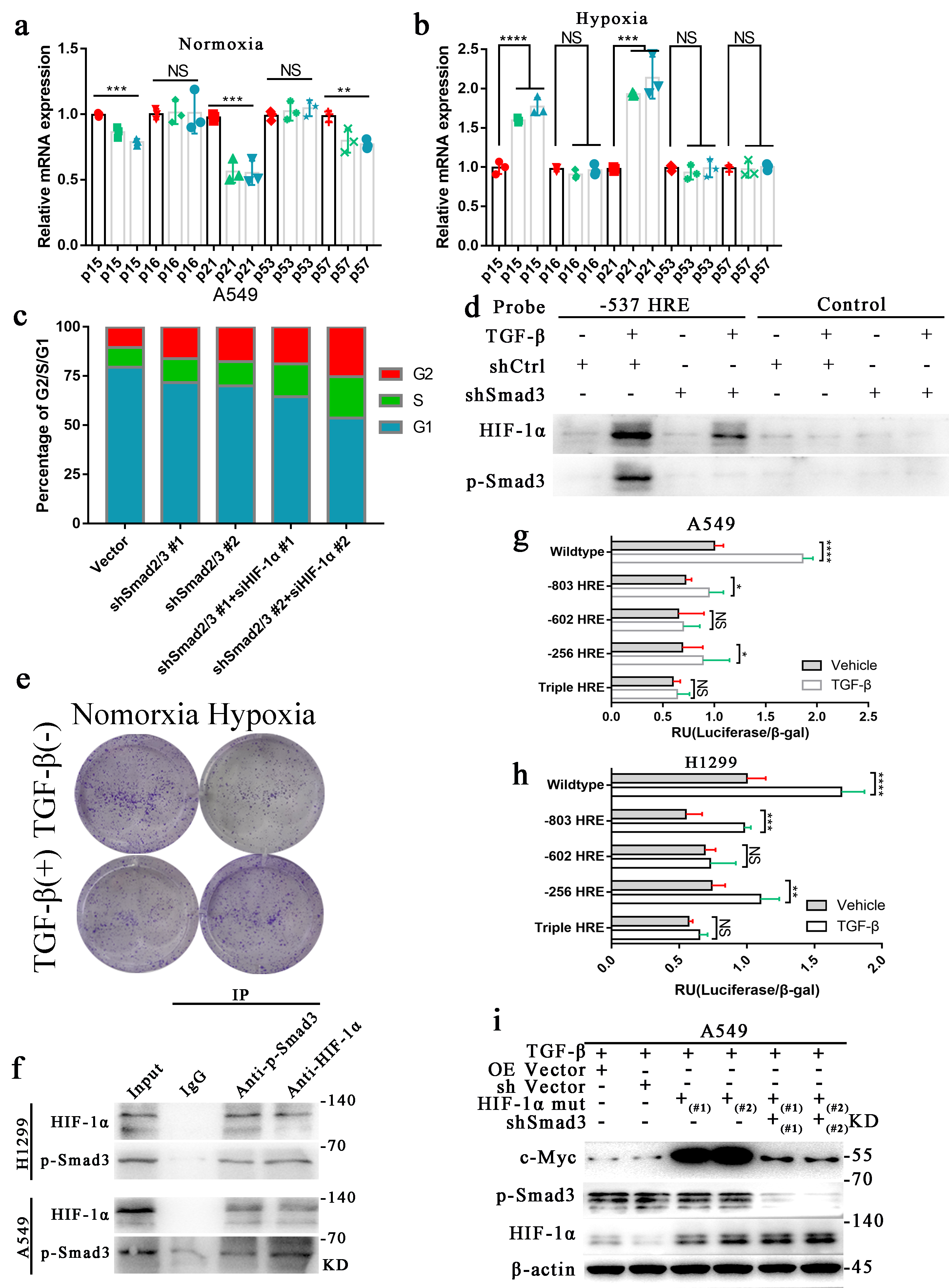

Supplement: Supplementary file 6 — Additional file 6: Supplementary Fig. 6. a, Expression intensity distribution of p21 based on IHC staining in lung adenocarcinoma, squamous cell carcinoma, and adjacent tissues. b-f, Based on TCGA data analysis, Kaplan–Meier survival analysis revealed that the prognosis of patients with high expression of CDKN1A (p21) (h) and PTBP1 (f) is worse; this was not demonstrated with high expression of Smad2 (b), c-Myc (c), and HNRNPA1 (e). g–k, Based on TCGA data analysis, expression of HIF-1α was positively correlated with the expression of Smad2 (h) and Smad3 (g), and expression of TGF-β was positively correlated with expression of c-Myc (i) and PKM (j) but negatively associated with CDKN1A (p21) (k). [file 13046_2021_2188_MOESM6_ESM.tif]
