# Supplementary material for: HIF-1α switches the functionality of TGF-β signaling via changing the partners of smads to drive glucose metabolic reprogramming in non-small cell lung cancer
Source: J Exp Clin Cancer Res. 2021 Dec 20;40:398. doi: 10.1186/s13046-021-02188-y (PMC8690885; doi:10.1186/s13046-021-02188-y)
Supplement: Supplementary file 7 — Additional file 7. [file 13046_2021_2188_MOESM7_ESM.docx]

**Supplementary table 1**

| **REAGENT or RESOURCE** | **SOURCE** | **IDENTIFIER** |
| --- | --- | --- |
| **Chemicals, Peptides, and Recombinant Proteins** | | |
| Recombinant Human TGF-β1 | BEIJING T&L BIOLOGICAL TECHNOLOGY | #TL-643 |
| Puromycin Dihydrochloride | Beyotime, Shanghai, China | ST551-10mg |
| Blasticidin S HCl | Beyotime, Shanghai, China | ST018-10mg |
| Immunoassay kit | Gene Tech, Shanghai, China | GK500710 |
| Protein A+G Agarose (Fast Flow, for IP) | Beyotime, Shanghai, China | P2055-2ml |
| PI cell cycle and apoptosis Kit | absin | abs50005 |
| Dual Luciferase® Reporter Assay System | Beyotime, Shanghai, China | RG027 |
| PCR Amplification Kit | TaKaRa | R011 |
| PrimeScript™ RT reagent Kit with gDNA Eraser | TaKaRa | RR047A |
| Hieff UNICON® Power qPCR SYBR Green Master Mix | YEASEN | 11195ES08 |
| **Antibodies** | | |
| HIF-1α (D1S7W) XP® Rabbit mAb | Cell Signaling Technology | #36169 |
| Anti-HIF-1α- ChIP Grade | Abcam | ab2185 |
| Smad3 Antibody | Abways Technology | CY5013 |
| Smad3 (C67H9) Rabbit mAb | Cell Signaling Technology | #9523 |
| Phospho-Smad3 (Ser423/425) (C25A9) Rabbit mAb | Cell Signaling Technology | #9520 |
| Smad2 (D43B4) XP® Rabbit mAb | Cell Signaling Technology | #5339 |
| Phospho-SMAD2 (Ser465/Ser467) (E8F3R) Rabbit mAb | Cell Signaling Technology | #18338 |
| HA-Tag (C29F4) Rabbit mAb | Cell Signaling Technology | #3724 |
| Flag-Tag Mouse Monoclonal Antibody | Abways Technology | AB0008 |
| Rabbit polyclonal to PKM2 | Abcam | ab137852 |
| PKM1 (D30G6) XP® Rabbit mAb | Cell Signaling Technology | #7067 |
| β-Actin Mouse Monoclonal Antibody | Beyotime, Shanghai, China | AF5001 |
| Rabbit monoclonal [EPR9048(B)] to PTBP1 | Abcam | ab133734 |
| Mouse monoclonal [9H10] to hnRNP A1 | Abcam | ab5832 |
| Rabbit polyclonal to hnRNP A2B1 | Abcam | ab31645 |
| Rabbit monoclonal [Y69] to c-Myc | Abcam | ab32072 |
| Rabbit monoclonal [EPR362] to p21 | Abcam | ab109520 |
| Rabbit polyclonal to p15 INK4b | Abcam | ab53034 |
| Rabbit polyclonal to Histone H3-Nuclear Marker and ChIP Grade | Abcam | ab1791 |
| **Oligonucleotides** | | |
| ENO1 | Sangon Biotech | N/A |
| F: AAAGCTGGTGCCGTTGAGAA  R: GGTTGTGGTAAACCTCTGCTC |  |  |
| HK1 | Sangon Biotech | N/A |
| F: GCTCTCCGATGAAACTCTCATAG  R: GGACCTTACGAATGTTGGCAA |  |  |
| HK2 | Sangon Biotech | N/A |
| F: GAGCCACCACTCACCCTACT  R: CCAGGCATTCGGCAATGTG |  |  |
| PKM2 | Sangon Biotech | N/A |
| F: ATGTCGAAGCCCCATAGTGAA  R: TGGGTGGTGAATCAATGTCCA |  |  |
| PKM1 | Sangon Biotech | N/A |
| F: TGAAGAACTTGTGCGAGCCTCAA  R: CCTGCCAGACTCCGTCAGAACTA |  |  |
| LDHA | Sangon Biotech | N/A |
| F: ATGGCAACTCTAAAGGATCAGC  R: CCAACCCCAACAACTGTAATCT |  |  |
| PDK1 | Sangon Biotech | N/A |
| F: CTGTGATACGGATCAGAAACCG  R: TCCACCAAACAATAAAGAGTGCT |  |  |
| MCT4 | Sangon Biotech | N/A |
| F: AGGTATCCTTGAGACGGTCAG  R: CAAGCAGGTTAGTGATGCCG |  |  |
| GLUT1 | Sangon Biotech | N/A |
| F: GGCCAAGAGTGTGCTAAAGAA  R: ACAGCGTTGATGCCAGACAG |  |  |
| PFK1 | Sangon Biotech | N/A |
| F: CCAGCCTGTGTAGTGAGCCTCT  R: CAGTGGAGCGAACAGCAGCATT |  |  |
| HIF1A | Sangon Biotech | N/A |
| F: AGTTCCGCAAGCCCTGAAAGC  R: GGCAGTGGTAGTGGTGGCATTA |  |  |
| SMAD2 | Sangon Biotech | N/A |
| F: GGGATGCTTCAGGTAGGACA  R: TCCGTCTCTTCCTGTGTCCT |  |  |
| PKM | Sangon Biotech | N/A |
| F: CGTGTTGTTCCTGTGCCGTGAT  R: CCTCCAGTCCAGCATTCCTCCTT |  |  |
| SMAD3 | Sangon Biotech | N/A |
| F: GATGGAGAGGCTTCCTGATG  R: TCTCTTTGCCAGGAATGCTT |  |  |
| ACTB | Sangon Biotech | N/A |
| F: TGACGTGGACATCCGCAAAG  R: CTGGAAGGTGGACAGCGAGG |  |  |
| CDKN1A (P21) | Sangon Biotech | N/A |
| F: TGTCCGTCAGAACCCATGC  R: AAAGTCGAAGTTCCATCGCTC |  |  |
| MYC | Sangon Biotech | N/A |
| F: AGCCACAGCATACATCCT  R: CGCACAAGAGTTCCGTAG |  |  |
| PTBP1 | Sangon Biotech | N/A |
| F: AGCGCGTGAAGATCCTGTTC  R: CAGGGGTGAGTTGCCGTAG |  |  |
| hnRNPA1 | Sangon Biotech | N/A |
| F: CAACTGATGAGAGCCTGAG  R: ACTCTTCCATCCACCTTGT |  |  |
| hnRNPA2B1 | Sangon Biotech | N/A |
| F: GAGGAGGAAGAGGAGGATAT  R: ATTGGACCGTAGTTAGAAGG |  |  |
| CDKN2B (P15) | Sangon Biotech | N/A |
| F: ACGGAGTCAACCGTTTCGGGAG  R: GGTCGGGTGAGAGTGGCAGG |  |  |
| CDKN2A (P16) | Sangon Biotech | N/A |
| F: CTCGTGCTGATGCTACTGAGGA  R: GGTCGGCGCAGTTGGGCTCC |  |  |
| TP53 (P53) | Sangon Biotech | N/A |
| F: CCTCAGCATCTTATCCGAGTGG  R: TGGATGGTGGTACAGTCAGAGC |  |  |
| CDKN1C (P57) | Sangon Biotech | N/A |
| F: AGATCAGCGCCTGAGAAGTCGT  R: TCGGGGCTCTTTGGGCTCTAAA |  |  |
| **shRNA Knockdown Sequences** |  |  |
| SMAD3 targeting siRNA sequence:  Sh1：CCAGTGCATATGCAATGTATA  Sh2：CGGCTATATTGGTTTATGTAGT | Hanyin Biotechnology | #LV201709-233 |
| SMAD2 targeting siRNA sequence:  Sh1：CAAGTACTCCTTGCTGGATTG  Sh2：CCAGTAATAGTTGCATTGATA | Hanyin Biotechnology | #LV201709-234 |
| HIF1A targeting siRNA sequence:  Sh1：TATGCACTTTGTCGCTATTAA  Sh2：GCCGCTCAATTTATGAATATT | Hanyin Biotechnology | #LV201712-331 |
| **siRNA Knockdown Sequences** |  |  |
| PKM2 targeting siRNA sequence:  Si1：GCCAUAAUCGUCCUCACCA  Si2：CCAUAAUCGUCCUCACCAA | Hanyin Biotechnology |  |
|  |  |  |
| **Software and Algorithms** | | |
| Graphpad Prism 8 | Graphpad Software,Inc. | https://www.graphpad.com/scientific-software/prism/ |
| ImageJ | National Institute of Health | https://imagej.nih.gov/ij/download.html |
| Photoshop CS6 | Adobe | https://www.adobe.com/products/photoshop/free-trial-download.html# |
| SPSS Statistics v 24.0 | IBM | https://www.ibm.com/support/pages/downloading-ibm-spss-statistics-24 |
| Rstudio & R software | R Core Team | https://rstudio.com/products/rstudio/download/ |
| ModFit LT version 5.0 | Verity Software House | http://www.vsh.com/vshstore/shopexd.asp?id=92&bc=no |
| Circlize | (Gu et al., 2014) | https://jokergoo.github.io/circlize_book/book/ |
| **Experimental Models: Cell Lines** | | |
| Human: A549 | Chinese Academy of Sciences | N/A |
| Human: H1299 | Chinese Academy of Sciences | N/A |
| Human: HEK293T | Chinese Academy of Sciences | N/A |
| **Biological Samples** | | |
| Human lung tissue chip | Zhongshan Hospital, Fudan university | N/A |
| Human lung tissue paraffin section | Zhongshan Hospital, Fudan university | N/A |
| Paraffin section of subcutaneous tumor | Central lab of Zhongshan hospital, Fudan university | N/A |
